# Somato-Dendritic Localization and Signaling by Leptin Receptors in Hypothalamic POMC and AgRP Neurons

**DOI:** 10.1371/journal.pone.0077622

**Published:** 2013-10-29

**Authors:** Sangdeuk Ha, Scott Baver, Lihong Huo, Adriana Gata, Joyce Hairston, Nicholas Huntoon, Wenjing Li, Thompson Zhang, Elizabeth J. Benecchi, Maria Ericsson, Shane T. Hentges, Christian Bjørbæk

**Affiliations:** 1 Department of Medicine, Division of Endocrinology and Metabolism, Beth Israel Deaconess Medical Center and Harvard Medical School, Boston, Massachusetts, United States of America; 2 Electron Microscopy Facility, Harvard Medical School, Boston, Massachusetts, United States of America; 3 Department of Biomedical Sciences, Colorado State University, Fort Collins, Colorado, United States of America; Institut National de la Santé et de la Recherche Médicale (INSERM U901), France

## Abstract

Leptin acts via neuronal leptin receptors to control energy balance. Hypothalamic pro-opiomelanocortin (POMC) and agouti-related peptide (AgRP)/Neuropeptide Y (NPY)/GABA neurons produce anorexigenic and orexigenic neuropeptides and neurotransmitters, and express the long signaling form of the leptin receptor (LepRb). Despite progress in the understanding of LepRb signaling and function, the sub-cellular localization of LepRb in target neurons has not been determined, primarily due to lack of sensitive anti-LepRb antibodies. Here we applied light microscopy (LM), confocal-laser scanning microscopy (CLSM), and electron microscopy (EM) to investigate LepRb localization and signaling in mice expressing a HA-tagged LepRb selectively in POMC or AgRP/NPY/GABA neurons. We report that LepRb receptors exhibit a somato-dendritic expression pattern. We further show that LepRb activates STAT3 phosphorylation in neuronal fibers within several hypothalamic and hindbrain nuclei of wild-type mice and rats, and specifically in dendrites of arcuate POMC and AgRP/NPY/GABA neurons of *Leprb* ^*+/+*^ mice and in *Leprb* ^*db/db*^ mice expressing HA-LepRb in a neuron specific manner. We did not find evidence of LepRb localization or STAT3-signaling in axon-fibers or nerve-terminals of POMC and AgRP/NPY/GABA neurons. Three-dimensional serial EM-reconstruction of dendritic segments from POMC and AgRP/NPY/GABA neurons indicates a high density of shaft synapses. In addition, we found that the leptin activates STAT3 signaling in proximity to synapses on POMC and AgRP/NPY/GABA dendritic shafts. Taken together, these data suggest that the signaling-form of the leptin receptor exhibits a somato-dendritic expression pattern in POMC and AgRP/NPY/GABA neurons. Dendritic LepRb signaling may therefore play an important role in leptin’s central effects on energy balance, possibly through modulation of synaptic activity via post-synaptic mechanisms.

## Introduction

Leptin, an adipocyte-derived hormone, acts on the central nervous system to regulate energy balance, glucose metabolism and neuroendocrine actions by activating the long signaling form of the leptin receptor (LepRb) [1-4]. Among several leptin-responsive brain regions, the arcuate nucleus of the hypothalamus (Arc) serves an important role in mediating these leptin actions. Within the Arc there are several distinct subsets of key LepRb-expressing neurons, including the anorexigenic pro-opiomelanocortin (POMC)-producing neurons and the orexigenic Agouti-related peptide (AgRP)/Neuropeptide Y (NPY)/γ-aminobutyric acid (GABA)-producing neurons [5-7]. Recent studies employing optogenetics and pharmacogenetic methods to modulate the activity of those neurons *in vivo* further demonstrate potent effects on feeding behavior [8-10]. Leptin stimulates c-Fos expression and increases firing rates of POMC neurons [11,12]. In contrast, the action potential frequency of AgRP/NPY/GABA neurons is depressed by leptin [13,14]. Consistent with these actions, fasting, a state of low circulating leptin concentrations, leads to opposite effects on action potentials on these two neuronal populations [15,16]. Mice lacking leptin receptors only in POMC or AgRP neurons exhibit increased fat mass accumulation, demonstrating that both groups of cells are required for maintenance of normal body weight by leptin [17,18]. In addition, diet-induced obesity in rodents is associated with hyperleptinemia and with impaired LepRb signaling in arcuate neurons, including POMC and AgRP/NPY/GABA cells, supporting the notion that diet-induced defects in LepRb signaling within these neurons may play a primary role in the development of obesity [4,19-21].

Leptin has structural homology to cytokines and the leptin receptor bears strong sequence similarity to the class I cytokine receptor super family [22,23]. Moreover, the signaling capabilities of LepRb show direct similarities to that of the signaling subunits of the interleukin 6 (IL-6)-cytokine receptor, leukemia inhibitory factor receptor (LIFR), and ciliary neurotrophic factor receptor (CNTFR) [24-27]. In particular, LepRb activates intracellular signaling actions through cytokine-receptor-like pathways including the canonical JAK2-STAT3 pathway [28,29]. Leptin-dependent phosphorylation of the STAT3 transcription factor is a highly specific CNS cellular marker for identification of LepRb-expressing neurons *in vivo* [19,30] and STAT3 activation by LepRb is critically important for normal energy balance regulation [31,32]. 

LepRb is required for leptin-mediated cell-autonomous effects on membrane potentials and axonal firing of hypothalamic neurons [33,34]. Furthermore, studies have shown modulation of excitatory Ca^2+^-currents via post-synaptic LepRb-dependent mechanisms [35,36]. Yet other evidence suggests that pre-synaptic actions of LepRb can modulate glutamate release onto dopamine neurons in the ventral tegmental area (VTA) [37] and influence GABA-release from AgRP/NPY/GABA neurons onto hypothalamic POMC neurons [12]. 

Despite progress in the understanding of neuronal leptin receptor signaling and of leptin’s metabolic actions via arcuate POMC and AgRP/NPY/GABA neurons, direct evidence of the sub-cellular localization of LepRb within soma and neuronal fiber compartments is not known. We show here that LepRb is expressed and signals in somato-dendritic compartments of both groups of neurons. In addition, we present evidence of LepRb signaling in close proximity to post-synaptic structures on dendritic shafts. These studies suggest that leptin receptor signaling in dendrites may be important for leptin’s effect on energy balance, possibly by modulating neuronal excitability through post-synaptic signaling mechanisms. 

## Materials and Methods

### Ethics Statement

Animal care and procedures were approved by the Institutional Animal Care and Use Committee at Beth Israel Deaconess Medical Center. 

### Mice and Rats

Mice and rats were housed at 22–24°C using a 14 hr light/10 hr dark cycle with standard chow diet (Teklad F6 Rodent Diet 8664, Harlan Teklad, Madison, WI). Transgenic mice with cre-dependent activation of HA-tagged LepRb expression (*HA-Leprb flox*)(prior name: *HA-ObRb STOP*) were described previously [38]. Briefly, *Pomc-cre* [17] and *Agrp-ires-cre* mice [39] were kindly supplied by Dr. B. Lowell (BIDMC, Boston, MA) and *Pomc-EGFP* mice were described earlier [12]. *EGFP flox* (Z/EG) reporter mice (B6.129(Cg)-Tg(CAG-Bgeo/GFP)21Lbe/J) were purchased from Jackson Laboratories (Bar Harbor, ME)[40]. *Lepr* ^*db/db*^;*Pomc-cre;HA-Leprb flox* and *Lepr* ^*db/db*^;*Agrp-ires-cre;HA-Leprb flox* mice that selectively express HA-LepRb in POMC or AgRP neurons respectively, were generated by crossing the appropriate *cre/lox* lines with *Lepr* ^*db/+*^ mice, as we have described previously [38]. The above animals were on mixed genetic backgrounds (FVB; C57BL/KsJ; C57BL/6J; 129). Adult male wild type C57BL/6J mice and male Sprague Dawley rats were purchased from Jackson laboratories. 

### Materials

Murine leptin was purchased from Dr. A.F. Parlow (National Hormone & Peptide Program, Torrance, CA). Supplies for immunohistochemistry (IHC) were purchased from Sigma-Aldrich (St. Louis, MO), apart from the ABC Vectastain Elite kit which was purchased from Vector Laboratories (Burlingame, CA) and the tyramide-signal-amplification kit (TSA) which was from Perkin Elmer (Waltham, MA). The phospho-specific-(Y705)-STAT3 rabbit antibody was from New England Biolabs (Beverly, MA) and the anti-HA mouse antibody from Covance Inc. (Berkeley, CA). The rabbit anti-POMC precursor (against amino residues 27-52) antiserum was obtained from Phoenix Pharmaceuticals (Burlingame, CA), the rabbit anti-β-endorphin antibody was a kind gift from Dr. Ronnekleiv (Oregon Health and Science University, Portland, OR)[41] and the sheep anti-α-MSH antiserum was from Chemicon International, Inc. (Temecula, CA). The chicken polyclonal anti-GFP and the mouse monoclonal anti-PSD95 antibodies were purchased from Abcam (Cambridge, MA). Biotinylated secondary antibodies were from Jackson Immunology Research Laboratories (West Grove, PA) and secondary fluorescent immunoglobulin conjugates were obtained from Molecular Probes (Eugene, OR). The plasmid encoding murine LepRb was described earlier [42,43]. The HA-LepRb expression vector was created by standard procedures and ultimately cloned into the mammalian expression vector pcDNA3.1 from Invitrogen (San Diego, CA). As we have described earlier, the DNA sequence encoding the 9 amino acid HA tag was inserted after codon 46 of the murine LepRb cDNA [38]. The EGFP expression vector was from Clontech Laboratories, Inc. (Mountain View, CA).

### Immunohistochemistry (IHC)

Transcardiac perfusion and fixation with paraformaldehyde, removal of brains, postfixation, and cryoprotection were performed as we have generally described previously [19,21,38]. Brains were cut in 25-35 µm thick coronal sections, collected in 4-5 series, and stored at -20°C until further use. For non-fluorescent light microscopy, free-floating brain sections were incubated with biotinylated secondary antibodies, followed by avidin-biotin complex labeling, and developed with nickel-diaminobenzidine (DAB), generating a brown-black precipitate for analyses by light microscopy (LM)[19,30]. For fluorescence microscopy, the free-floating sections were incubated with fluorescent-labeled secondary antibodies [44,45]. In the case of HA immunofluorescence, TSA procedures were applied to enhance sensitivity (Perkin Elmer). DAB results were visualized using bright-field light and photomicrographs captured with a digital AxioCam camera (Carl Zeiss, Thornwood, NY) mounted on a Zeiss microscope (Axioscope2) or a Nikon Eclipse 80i equipped with a Nikon DS-U1 digital camera (Nikon Instruments Inc., Melville, NY). Fluorescent results were captured and analyzed by confocal laser scanning microscopy (CLSM) using a Zeiss LSM510 system and the LSM Image Browser 4.2.0.121 free-ware. Adobe Photoshop software (Adobe, San Jose, CA) was used to crop images and to change file types for publication.

### Electron Microscopy (EM)

For transmission EM, mouse brains were first fixed via transcardiac perfusion with saline followed by 1.0% formaldehyde and 1.25% EM grade glutaraldehyde (TedPella, Redding, CA) in phosphate buffer, pH 7.4 (PB). Brains were removed from the skull and post-fixed in 2.0% formaldehyde and 2.5% glutaraldehyde in PB at 4°C overnight. Following embedding in 3% LMP agarose, 100 µm thick coronal sections were cut on a vibratome and stored in 2.0% formaldehyde and 2.5% glutaraldehyde in PB at 4°C until further use (method modified from [46]). For pre-embedding EGFP immuno-EM, free floating sections were blocked with goat serum and incubated with anti-GFP antibodies overnight at room temperature [44]. For pre-embedding pSTAT3 immuno-EM, free floating brain sections were pretreated with NaOH/H_2_O_2_, glycine and SDS, blocked with goat serum, and incubated with anti-pSTAT3 antibodies overnight at room temperature, as described [30]. For DAB staining, the sections were incubated with biotinylated secondary antibodies, subjected to avidin-biotin complex labeling, and developed with nickel-diaminobenzidine (DAB) as described above under IHC. For pre-embedding pSTAT3 immuno-Gold-EM, free floating sections were first pretreated and treated with the anti-pSTAT3 antibodies as above, washed, and then incubated with anti-rabbit IgG conjugated to 1 nm gold particles (Amersham Biosciences Inc). Post-fixation, tissue embedding, cutting, placement on grids, and counter staining were performed by the Electron Microscope Facility at Harvard Medical School.

Immuno-stained tissue sections were silver enhanced with the InteSE kit (Amersham Biosciences), then postfixed in 0.5 % osmium tetroxide for 15 minutes, rinsed in water, dehydrated in graded ethanol solutions, transferred to propylene oxide, and embedded in epoxy resin (Epon-Araldite, EMS, Hatfield, PA) between two layers of Aclar plastic (Epon-Araldite, EMS, Hatfield, PA). After the plastic was polymerized at 60°C for 48 hrs, the area of the arcuate hypothalamic nucleus was dissected out and re-mounted on a plastic block for sectioning. Ultrathin 50 nm thick sections were then cut on an Ultracut-S ultramicrotome (Reichert Technologies, Depew, NY) and mounted on copper grids. Before examination, grids with DAB stained sections were negatively stained with 1% uranyl acetate, while grids with IgG gold labeled sections were negatively stained with both uranyl acetate and lead citrate. Finally, grids were examined in a TecnaiG2 Spirit BioTWIN and photomicrograph images were recorded with an AMT 2k CCD camera at the Electron Microscope Facility, Harvard Medical School. For serial EM, consecutive sections were aligned and selected structures were reconstructed using the 3D reconstruction software, Reconstruct (http://synapses.clm.utexas.edu/tools/reconstruct/reconstruct.stm) [47,48]. 

Ultrafine structures in the central nervous system were identified as described by Peters et al. [49] and Stuart et al. [50]. Briefly, neuronal cell bodies were identified by their large cytoplasmic space when compared with dendritic shafts, the presence of multiple intracellular compartments, including the nucleus and organelles, and although not essential because of the orientation of the sections, the protrusion of large proximal dendrites from their membrane. Dendritic shafts were identified by having a cross section diameter of 0.5-2 µm with a relatively irregular shape, and often by the presence of a regular array of microtubules. Axon fibers were defined as either myelinated or not, smaller cross section diameter compared to dendritic shafts and tending to maintain a consistent convex cross section. In addition, axon fibers typically exhibit a denser and more organized array of microtubules relative to dendritic shafts. Glial fibers are highly irregular in shape, often containing glycogen granules, and further differ from dendritic shafts and axons by not having a regular array of microtubule fibers. Spines were defined as their relative small size (typically ~1-2 µm length) compared to the dendritic shaft and as any protrusion from the dendritic shaft that is not a dendrite branch. In addition, spines are usually devoid of mitochondria and microtubules. Asymmetric synapses form synaptic structures that are distinguished by a thickened, postsynaptic density (PSD) and a minor presynaptic density. In contrast, in symmetric synapses the pre-and postsynaptic membranes are typically more parallel than the surrounding non-synaptic membrane, and the synapse has a less prominent postsynaptic density that is similar in thickness to the pre-synaptic density.

### Primary Hypothalamic and Hippocampal Neuronal Cultures

NeuroPure E18 primary rat hypothalamus cells (#N400200) and Neuropure E18 primary rat hippocampal cells (#N100200) were purchased from Genlantis (San Diego, CA) and were dissociated, plated and grown in 24 well dishes on poly-D-lysine-coated coverslips as generally directed by the manufacturer. After 4-7 days in vitro (DIV), neurons were transfected with LepRb expression vectors, or co-transfected with HA-LepRb and EGFP expression vectors using NeuroFECT (Genlantis) or LipoFectamine 2000 (Invitrogen) according to the manufacturer’s instructions. One to two days later, neurons were serum-deprived for 3-12 hours and treated with 100 nM recombinant leptin or vehicle control solution. Cells were then fixed and the coverslips were subjected to pSTAT3- or HA-immunocytochemistry (ICC) as we have generally described previously [30,38]. 

### In situ Hybridization

Free-floating brain sections were generated as described for IHC (above) and processed for *in situ* as described earlier [21]. Briefly, digoxigenin (DIG) labeled RNA probes were generated by PCR amplification of mouse hypothalamic cDNA utilizing sets of POMC or NPY primers that included SP6 or T7 end-sequences. Specifically, for POMC we used a 628 base long probe (bases 147-775 of POMC mRNA (NCBI: NM_008895)) and for NPY a 308 base long (bases 67-376 of NPY mRNA (NCBI: NM_023456)). Purified PCR products were then subjected to *in vitro* RNA synthesis of sense or antisense RNA’s using appropriate SP6 or T7 polymerases and DIG labeling mix. Briefly, sections were mounted on Superfrost Plus slides (Fisher, Hampton, NH) and hybridized overnight with the DIG-labeled mouse *Pomc* anti-sense RNA probe or with the DIG-labeled mouse *Npy* anti-sense RNA probe, both at 0.6 µg/ml at 60°C. All brain sections were washed twice in 0.2X SSC at 60°C, blocked in PBS with 10% bovine serum, and reacted with anti-DIG antibodies fused to alkaline phosphatase (Roche, Nutley, NJ)(1:5000, 10% serum, 2 hours at room temperature). Sections were washed and incubated with alkaline phosphatase substrate (NBT/BCIP, Roche, Nutley, NJ) producing a color precipitate. The reaction was stopped by addition of EDTA.

## Results

### Leptin activates STAT3 phosphorylation in neuronal fiber processes in hypothalamic nuclei of mice and rats

When we first developed the immunohistochemical (IHC) assay for phosphorylated STAT3 (pSTAT3) in brain sections from leptin-treated rodents [19,30] we noted pSTAT3 immunoreactivity (IR) in many neuronal fiber processes in addition to the expected nuclear staining. This observation and the relationship to LepRb sub-cellular localization was further investigated here. 

**Figure 1:**
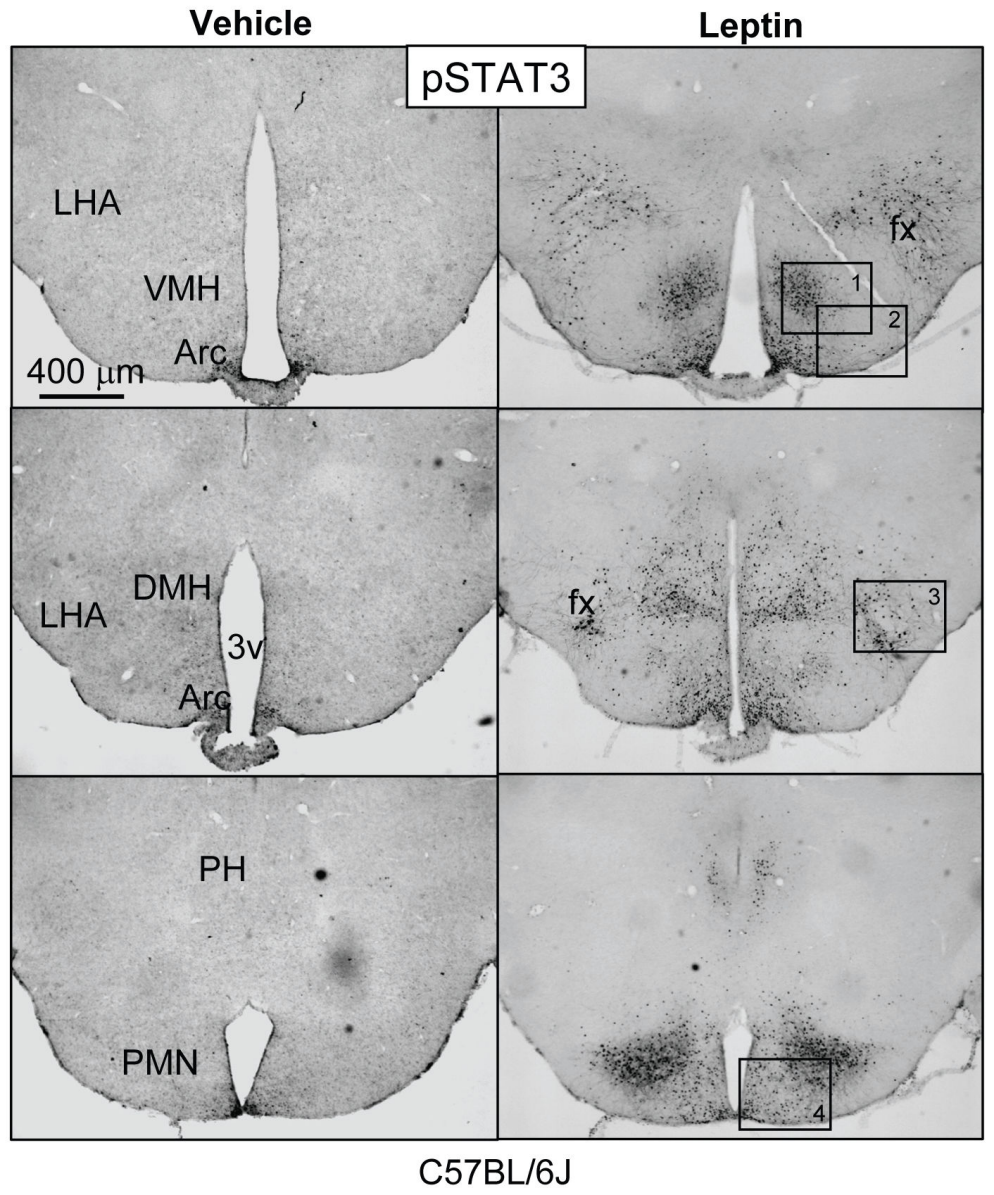
Leptin activates STAT3 phosphorylation in hypothalamic nuclei of C57BL/6J mice. Shown are light microscopy (LM) images of phospho-STAT3 immunoreactivity (IR) (DAB) in coronal brain sections of the mediobasal hypothalamus from 8-12 weeks old wild type C57BL/6J male mice. Animals were given vehicle or leptin (5 mg/kg, ip) and sacrificed after 30 minutes. Weak pSTAT3 IR is detected in the arcuate nucleus of the vehicle treated animals. In contrast, robust pSTAT3 IR is found in the LHA, VMH, DMH, Arc and PMN of the leptin-treated mouse. Three matched rostral-to-caudal sections are shown for both treatment groups. Boxes 1-4 are magnified in Figure 2. LHA: lateral hypothalamic area; VMH: ventro-medial hypothalamic nucleus; DMH: dorso-medial hypothalamic nucleus; Arc: arcuate hypothalamic nucleus; PMN: premammillary hypothalamic nucleus; fx: fornix, PH: posterior hypothalamic region; 3v: 3^rd^ ventricle.

**Figure 2:**
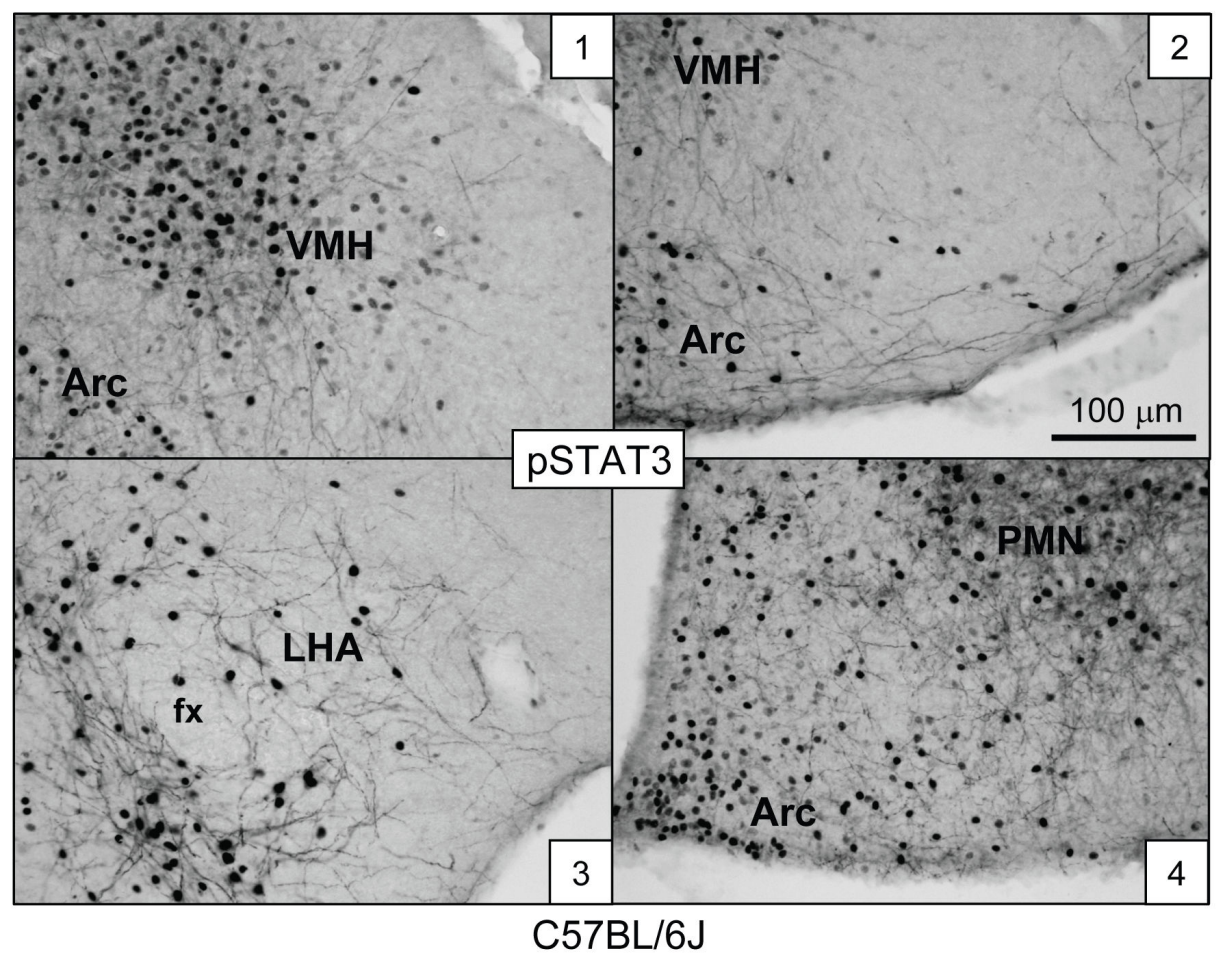
Leptin activates STAT3 phosphorylation in neuronal processes within hypothalamic nuclei of C57BL/6J mice. High-magnification LM microphotographs of numbered boxes in Figure 1. Many pSTAT3 IR neuronal fibers are observed in all nuclei. LHA: lateral hypothalamic area; VMH: ventro-medial hypothalamic nucleus; DMH: dorso-medial hypothalamic nucleus; Arc: arcuate hypothalamic nucleus; PMN: premammillary hypothalamic nucleus; fx: fornix, 3v: 3^rd^ ventricle.

Figure 1 shows microphotographs captured by light microscopy (LM) of coronal brain sections of the medio-basal hypothalamus from a vehicle and a leptin-treated wild type mouse. While almost no pSTAT3 IR is present in the vehicle-treated animal, robust nuclear staining is observed in many nuclei known to express LepRb, including the LHA, VMH, DMH, Arc, PH and the PMN (Figure 1). In contrast to rats [51], pSTAT3 IR nuclei were not detected in the paraventricular hypothalamic nucleus (PVH) of leptin-treated mice (Figure S2). Phospho-STAT3 is an established cellular marker for first-order LepRb-expressing neurons and is not activated in second-order neurons [30]. In addition to the expected nuclear staining described above, pSTAT3 IR was also observed within neuronal fiber processes (Figure 2). These fibers were largely confined to each brain nucleus where LepRb is known to be present [52,53]. Similar fiber staining was also found within the nucleus of the solitary tract (NTS) in the caudal hindbrain (Figure S1). Because the pSTAT3 IR fibers did not extend much beyond each brain nucleus, STAT3 activation by leptin may be limited to dendritic fiber structures rather than axons which project far beyond each brain nucleus. In support of this possibility, pSTAT3 IR fibers were not found in the PVH of mice, a region known to receive dense axonal innervation from arcuate leptin-responsive neurons, including POMC and AgRP neurons (Figure S2)[6,54,55]. Similar anatomical patterns of STAT3 IR neuronal fibers were also seen in hypothalamic nuclei of leptin-treated Sprague Dawley rats, including the VMH, DMH and PMN (Figure S3). These data combined suggest that LepRb itself may be localized to neuronal fibers, possibly dendrites.

### Leptin activates STAT3 phosphorylation in soma and fiber processes of hypothalamic POMC neurons of mice

**Figure 3:**
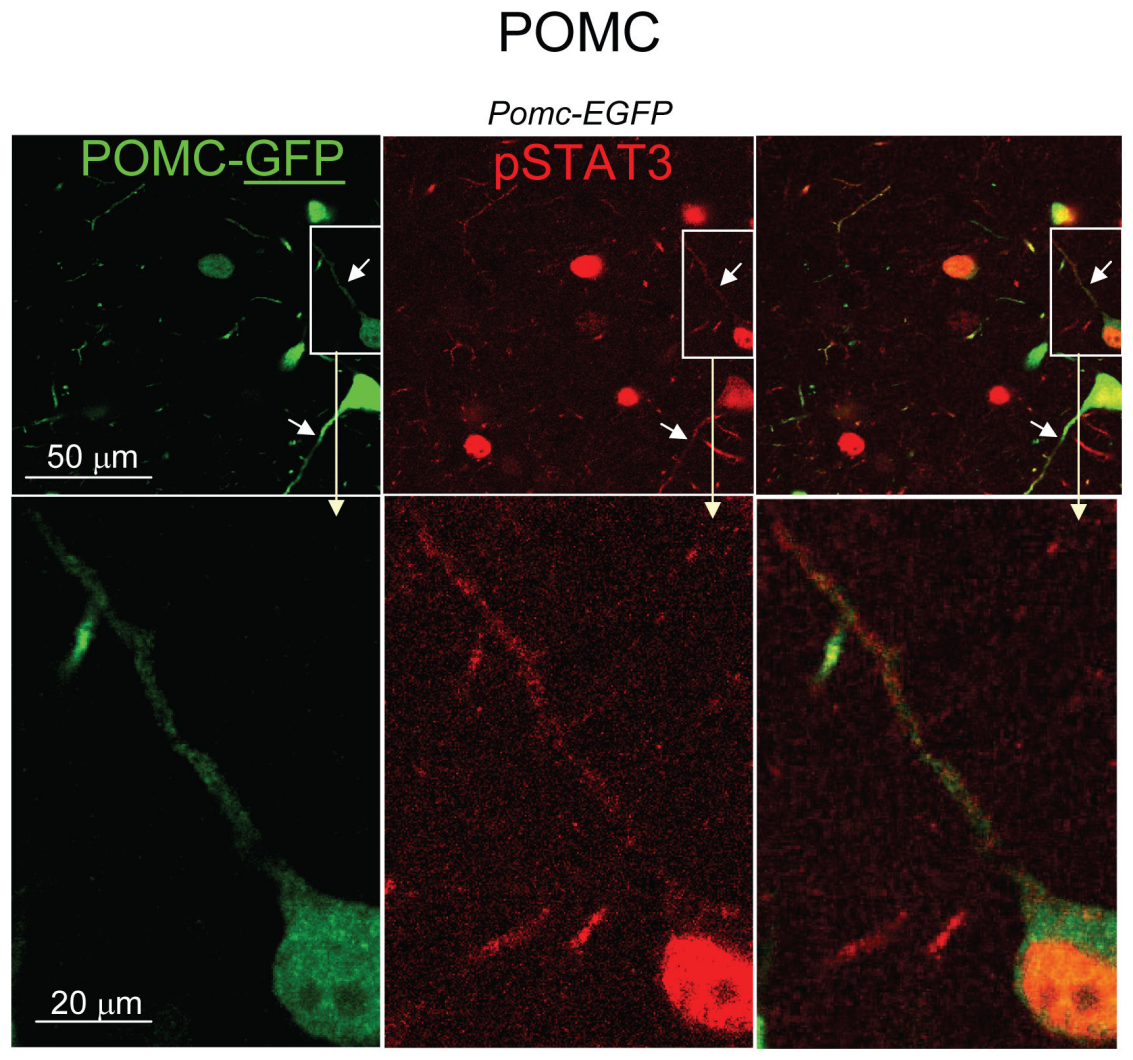
Leptin activates STAT3 phosphorylation in soma and processes of hypothalamic POMC neurons of mice. Top Row: *Pomc-EGFP* mice (6-10 wks of age) were given leptin (5 mg/kg, ip, 30 min). Shown is confocal laser scanning microscopy (CLSM) of fluorescent pSTAT3 IR (red) and EGFP-epifluorescence (green) in a brain section of the arcuate nucleus of the hypothalamus. pSTAT3 IR is observed both in the nucleus and cytoplasm, as well as in several fibers (arrows) of POMC neurons. Bottom Row: High magnification microphotographs of boxes in top panels. All images are single confocal planes.

To investigate whether STAT3 is activated in fiber structures of known LepRb-expressing hypothalamic neurons such as the POMC neurons, we next treated *Pomc-EGFP* mice with leptin and applied fluorescent-IHC and confocal laser scanning microscopy (CLSM) to hypothalamic brain sections. As shown in Figure 3, pSTAT3 IR is localized to the nucleus, cytoplasm and to proximal fibers of two POMC neurons (arrows). Similar data were obtained from the Arc of leptin-treated *Npy-hrGFP* mice (not shown). POMC (and AgRP/NPY/GABA) neurons might therefore serve as a suitable model system to investigate the sub-cellular fiber distribution of LepRb.

### HA-tagged LepRb is localized to intracellular vesicular structures and to the plasma membrane of POMC somata, and to proximal fibers of POMC neurons

To date, sufficiently sensitive and specific anti-LepRb antibodies capable of detecting endogenous LepRb proteins in the rodent brain have yet to be reported. We therefore employed a genetic strategy to express HA-tagged LepRb in mice thus facilitating detection with well characterized sensitive monoclonal anti-HA antibodies. The genetic design included introduction of two *loxP* sites flanking a transcriptional blocking sequence upstream of the HA-LepRb cDNA to allow HA-LepRb expression in a cre-dependent manner (e.g. expression of HA-LepRb in POMC neurons by crossing *HA-LepRb flox* mice with *Pomc-cre* mice). The detailed strategy was described earlier [38]. Successful co-expression of HA-LepRb and POMC polypeptides in *Pomc-cre;HA-Leprb flox* mice is shown by immunofluorescence-IHC and CLSM in Figure S4. 

**Figure 4:**
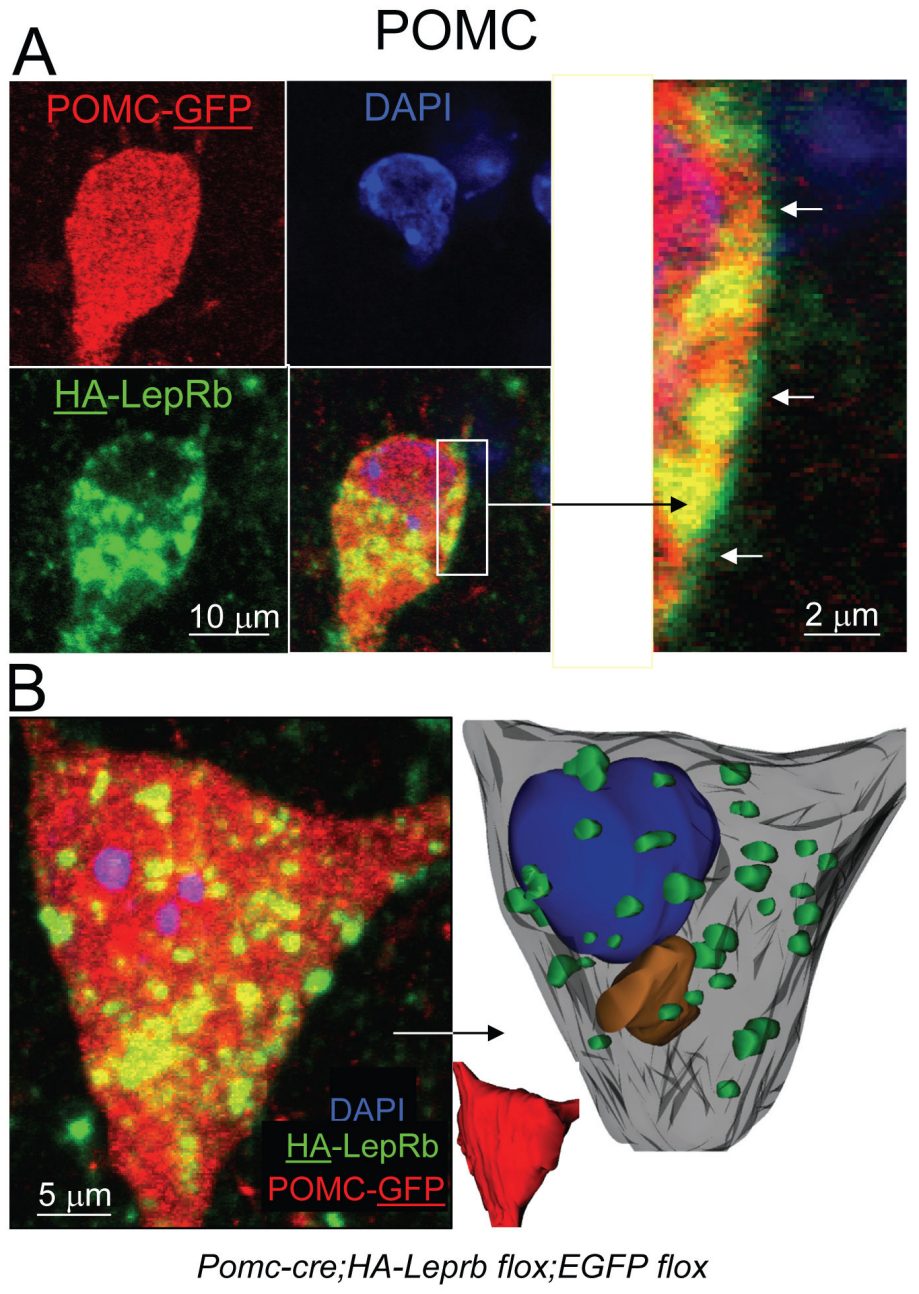
Localization of HA-tagged LepRb at the plasma membrane and cytoplasm of hypothalamic POMC neurons. **A**. CLSM of brain sections from a *Pomc-cre;HA-Leprb* *flox;EGFP* *flox* mouse. Shown is the soma of a hypothalamic POMC neuron expressing HA-LepRb (EGFP IR (red); HA IR (green); Nuclear DAPI (blue)). Within the cytoplasm, HA-LepRb is expressed in a vesicular/punctate pattern. Right: Enlargement of box area in A, depicting plasma membrane localization of HA-LepRb (arrows). Shown are single plane sections (0.42 µm). **B**. Left: CLSM was used to generate a collapsed Z-stack of the soma of a hypothalamic POMC neuron (EGFP IR (red)) expressing HA-LepRb (HA IR (green)). The Z-stack is ~16 µm deep comprising of 39 confocal sections. Right: The soma of the neuron is reconstructed in 3-dimensions using all 39 sections and the Reconstruct computer software. A non-transparent surface representation of the cell is shown in red (insert). A grey semi-transparent surface representation is also depicted. Blue is the nucleus (non-transparent). A number of vesicular-like structures that express HA-LepRb are shown in green. In addition, peri-nuclear localization of HA-LepRb in the presumed Golgi/trans-Golgi networks is represented in orange.

**Figure 5:**
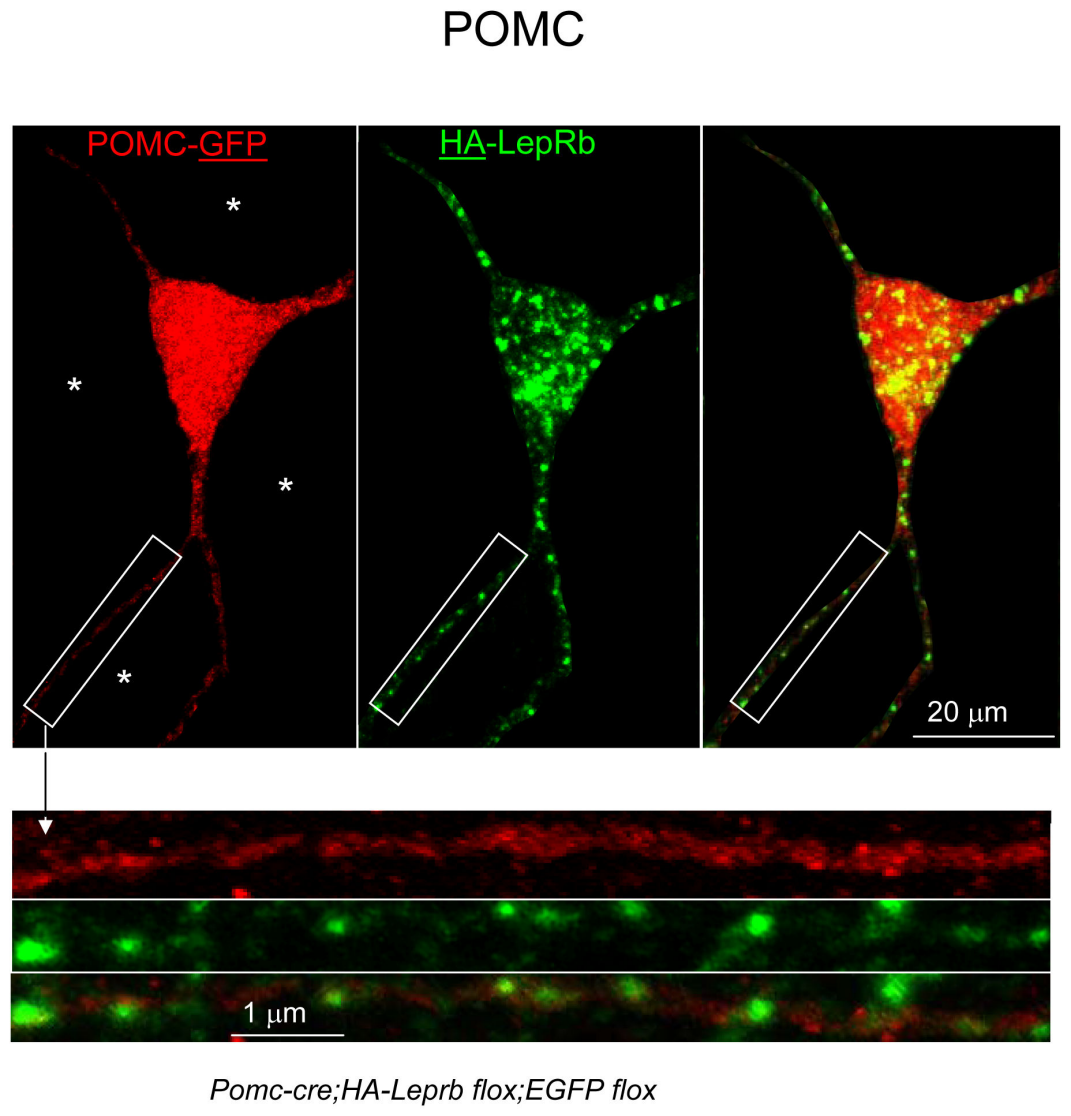
Localization of HA-tagged LepRb in proximal neuronal fibers of hypothalamic POMC neurons. CLSM was used to generate a collapsed Z-stack of a POMC neuron from a *Pomc-cre;HA-Leprb* *flox;EGFP* *flox* mouse. The soma and several proximal neuronal fibers can be seen. HA-LepRb is observed in a punctate pattern in three primary and one secondary fiber processes. Due to high background in the Z-stack and to more clearly identify the fibers, areas that not are associated with the POMC neuron have been blacked out (*). Bottom: Enlargement of boxes showing punctuate localization of HA-LepRb IR along a proximal POMC fiber.

 To enable investigation of possible LepRb localization in neuronal fibers, we created mice expressing HA-LepRb and EGFP in POMC soma and fiber processes (*Pomc-cre;HA-Leprb flox;EGFP flox*). Figure 4A shows the cell body of a POMC neuron (GFP IR) expressing HA-LepRb (HA IR). A large fraction of somatic HA-LepRb IR is found in vesicular-like structures within the cytoplasm. In addition, a minor fraction of HA-LepRb appears localized to the plasma membrane of the POMC soma. A POMC cell body is represented as a collapsed Z-stack and is reconstructed in 3-dimensions in Figure 4B. A non-transparent 3-D surface representation is shown in red. A semi-transparent representation of the POMC soma is also depicted. Green represents HA-LepRb localization in larger vesicular-like structures. A large fraction of HA-LepRb was detected in peri-nuclear regions, possibly representing Golgi and trans-Golgi networks (orange). Similar results were obtained from 3-D reconstruction of one additional POMC neuron (not shown). In Figure 5, proximal neurites extending from a POMC neuron are shown. HA-LepRb is found in a punctuate pattern of all primary and secondary fibers. These results indicate that HA-LepRb is at least localized to dendritic fibers of POMC neurons (i.e. only one axon per cell). 

In Figure S5A we show that HA-LepRb is expressed in proximal neurite fibers of primary hypothalamic neurons transfected with HA-LepRb plasmids. In addition, leptin activates STAT3 phosphorylation in neuronal fibers that contain the dendritic marker, PSD95 (Figure S5B), thus supporting a dendritic localization of LepRb.

### HA-LepRb is localized to dendrites, but not axons, of POMC neurons

**Figure 6:**
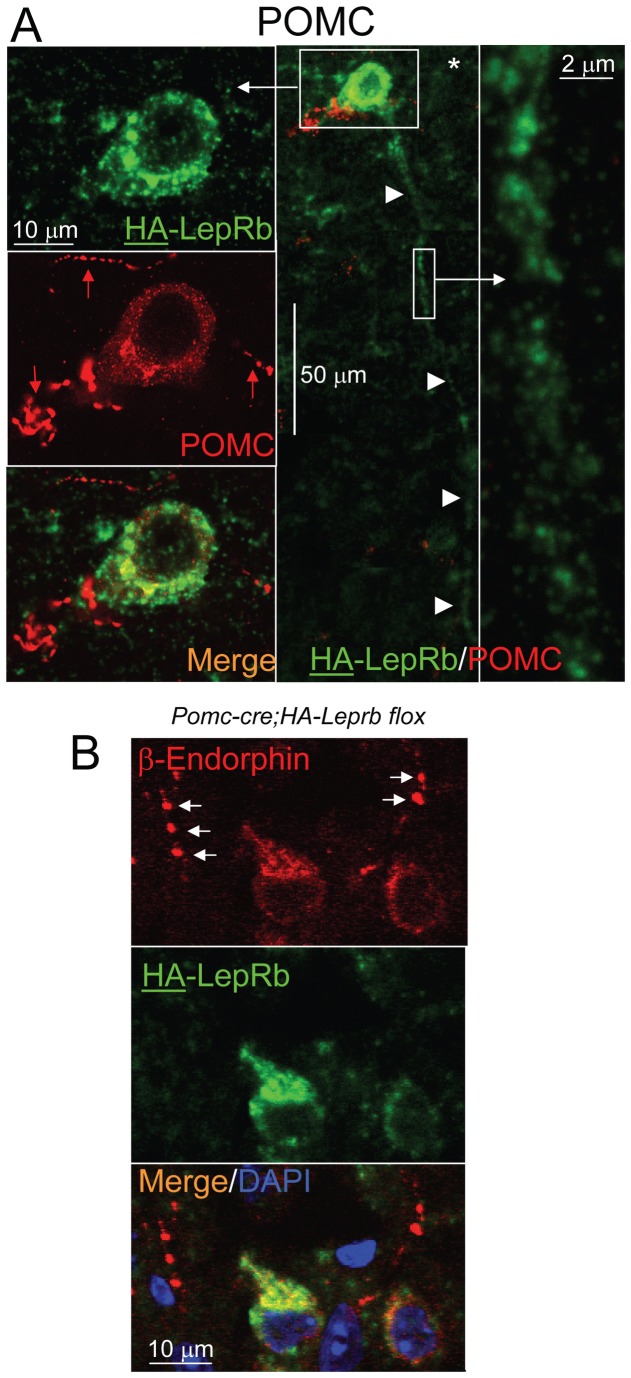
HA-LepRb is localized to dendrites, but not axons, of POMC neurons. **A**. Middle Image (*): CLSM was applied to depict a POMC neuron with a >200 µm long dendrite (4 arrow heads) in a brain section from a *Pomc-cre;HA-Leprb* *flox* mouse. POMC IR is shown in red and HA (LepRb) IR in green. To present the entire length of the dendrite, the image was merged from three different focal planes. Left Column: The POMC soma expresses both HA-LepRb and POMC polypeptides. None of the POMC axonal fibers (red arrows) in the field exhibit HA IR. Right Image. Magnification of box area depicting a fiber segment containing punctate HA-LepRb expression. POMC IR is not detectable in the fiber (dendrite). Shown are single confocal planes. **B**. Depicted are two POMC somata co-expressing β-endorphin IR (red) and HA-LepRb (HA IR (green)) in a brain section from a *Pomc-cre;HA-Leprb* *flox* mouse. Two nearby POMC IR-axonal fibers (arrows) do not express HA-LepRb. Nuclear DAPI is colored blue. These are single confocal planes.

We next aimed to determine if HA-LepRb is expressed in axonal fibers of POMC neurons by investigating possible co-localization with neuronal POMC axon-markers using antibodies against the POMC-polypeptide precursor or the POMC-derived β-Endorphin neuropeptide [54,56]. By fluorescence-IHC and CLSM we show in Figure 6A a POMC cell body with a long fiber (>200 µm) that expresses HA-LepRb (green) in a punctuate pattern. This fiber is relatively thick, does not show evidence of varicosities (boutons) and does not express the (red) POMC-polypeptide axonal marker (right enlargement), and is therefore likely a dendrite. In contrast, the POMC-precursor (red) can be found in the parent soma and in thin en-passant fibers with varicosities that are therefore likely axons (left column,). Furthermore, as shown in Figure 6B, HA-LepRb is not expressed in β-Endorphin positive fibers (white arrows) which exhibit bouton-like structures that are characteristic for axons. Based on these results, we conclude that the long-form leptin receptor is principally localized to the soma and dendrites, but not to axons of POMC neurons.

### Leptin activates STAT3 phosphorylation in dendrites, but not in α-MSH-containing axonal fibers of POMC neurons

We next examined if leptin can stimulate down-stream signal transduction in dendritic, but not axonal, cellular compartments of POMC neurons. In order to restrict signaling events to POMC neurons, we generated *Lepr* ^*db/db*^;*Pomc-cre;HA-Leprb flox* mice as we have described earlier [38]. These mice are 6-9 weeks old, obese and hyper-leptinemic [38]. In Figure S6, we applied IHC and LM to brain sections to first validate that leptin-treated *Lepr* ^*db/db*^;*Pomc-cre;HA-Leprb flox* mice exhibit pSTAT3 activation only in the arcuate hypothalamic regions that are consistent with the well known anatomical location of POMC neurons. As expected, STAT3 phosphorylation was not found in leptin-treated *Lepr* ^*db/db*^ control mice. Importantly, leptin-treated *Lepr* ^*db/db*^;*Pomc-cre;HA-Leprb flox* mice showed evidence of pSTAT3 IR in fiber processes (and POMC nuclei) (Figure S6), as we have shown in neuronal fibers of wild type mice (Figure 2) and in processes of POMC neurons of *Pomc-GFP* mice (Figure 3).

**Figure 7:**
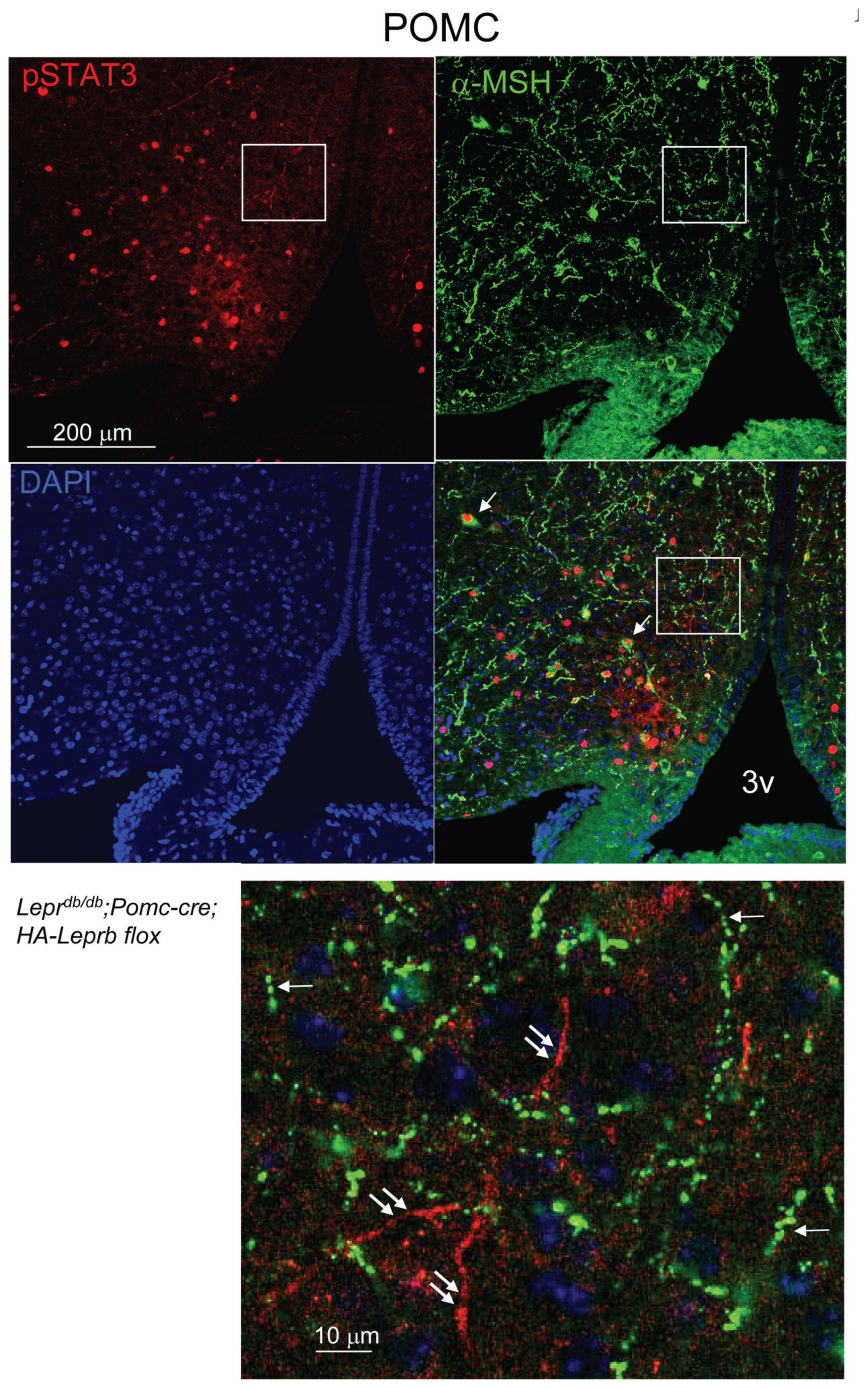
Leptin does not activate STAT3 phosphorylation in α-MSH-containing axonal fibers of POMC neurons. CLSM showing pSTAT3 IR (red) and α-MSH IR (green) and DAPI (blue) in the arcuate nucleus of a leptin-treated (5 mg/kg; 30 min) *Leprb^db/db^*;*Pomc-cre;HA-Leprb* *flox* mouse (8 weeks old). Top: Shown are single confocal planes. Examples of two POMC somata with nuclear pSTAT3 and cytoplasmic α-MSH are indicated with arrows in merged image. 3v; 3^rd^ ventricle. Bottom: Magnification of box area. Many α-MSH IR axonal fibers with boutons are found (single arrows). These do not co-express pSTAT3. In contrast, pSTAT3 IR fibers (double arrows) do not exhibit α-MSH IR and do not show presence of axonal boutons.

 In brain sections from leptin-treated *Lepr* ^*db/db*^;*Pomc-cre;HA-Leprb flox* mice we next applied fluorescent-IHC and CLSM to investigate fiber localization of pSTAT3 and the POMC-polypeptide-derived axonal marker, α-melanocyte stimulating hormone (α-MSH)[54,55,57,58]. As shown in Figure 7, pSTAT3 IR was observed in fiber processes that did not exhibit α-MSH IR. In contrast, α-MSH was localized to thin fibers with varicosities, consistent with being POMC axonal processes. Combined, these results indicate that leptin activates STAT3 phosphorylation in POMC dendrites, but not in axons, consistent with somato-dendritic localization of LepRb.

### Ultrastructural analyses of STAT3 phosphorylation by leptin in dendrites of hypothalamic POMC and AgRP/NPY neurons

To further validate and investigate LepRb localization and signaling in dendrites at the ultrastructural level of hypothalamic neurons, we applied immuno-electron microscopy (EM) methodology. Multiple attempts unfortunately failed to detect specific signals for HA-LepRb by immuno-HA EM, possibly due to antigen interference by glutaraldehyde in the EM fixative. We therefore focused these studies on investigations of the ultrastructural sub-cellular localization of leptin-induced STAT3 phosphorylation, as pSTAT3 immunostaining was not negatively affected by glutaraldehyde. These analyses were expanded to include investigations of the localization of leptin-induced pSTAT3 in AgRP neurons. To this end, brain sections from leptin-treated *Lepr* ^*db/db*^;*Pomc-cre;HA-Leprb flox* mice and *Lepr* ^*db/db*^;*AgRP-ires-cre;HA-Leprb flox* mice were subjected to pSTAT3 immuno-EM. Validation of proper anatomical localization of pSTAT3 IR consistent with the known location (ventro-medial Arc) of AgRP neurons in *Lepr* ^*db/db*^;*AgRP-ires-cre;HA-Leprb flox* mice is shown by IHC and LM in Figure S7. Of note, *Lepr* ^*db/db*^;*AgRP-ires-cre;HA-Leprb flox* mice (6-9 weeks old) are nearly as obese and hyperleptinemic as *Lepr* ^*db/db*^ controls (not shown). As in POMC fibers of *Lepr* ^*db/db*^;*Pomc-cre;HA-Leprb flox* mice, pSTAT3 IR was also observed in AgRP neuronal fibers of *Lepr* ^*db/db*^;*AgRP-ires-cre;HA-Leprb flox* mice at the LM level (Figure S7).

**Figure 8:**
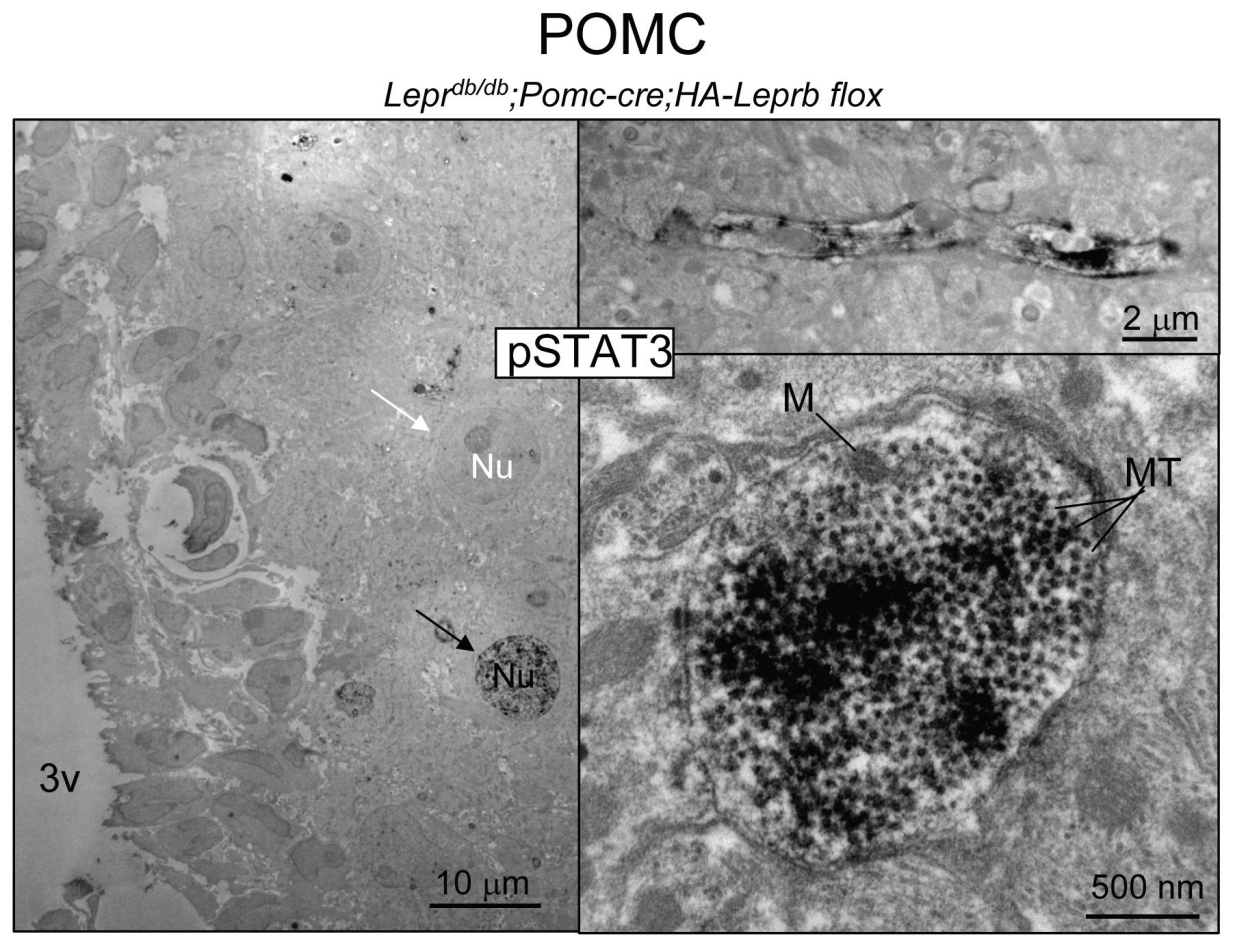
Activation of STAT3 phosphorylation by leptin in dendrites of hypothalamic POMC neurons by immuno-electron microscopy (EM). Immuno-EM for pSTAT3 (DAB) in a leptin-treated (4 mg/kg ip; 10 min) *Lepr^db/db^*;*Pomc-cre;HA-LepRb* *flox* mouse. Left image: EM scope magnification is 440x showing an overview of the medial arcuate hypothalamic region including the ependymal cells lining the 3rd ventricle (3v). A pSTAT3 IR neuronal nucleus is marked with a black arrow (Nu) and a pSTAT3-negative nucleus is labeled with a white arrow (Nu). Top Right: pSTAT3 IR dendrite in the horizontal plane (EM @ 1,200x). Bottom Right: Cross-section of pSTAT3 IR dendrite (EM @ 30,000x). A mitochondrion (M) and arrays of microtubules (MT) are seen.

**Figure 9:**
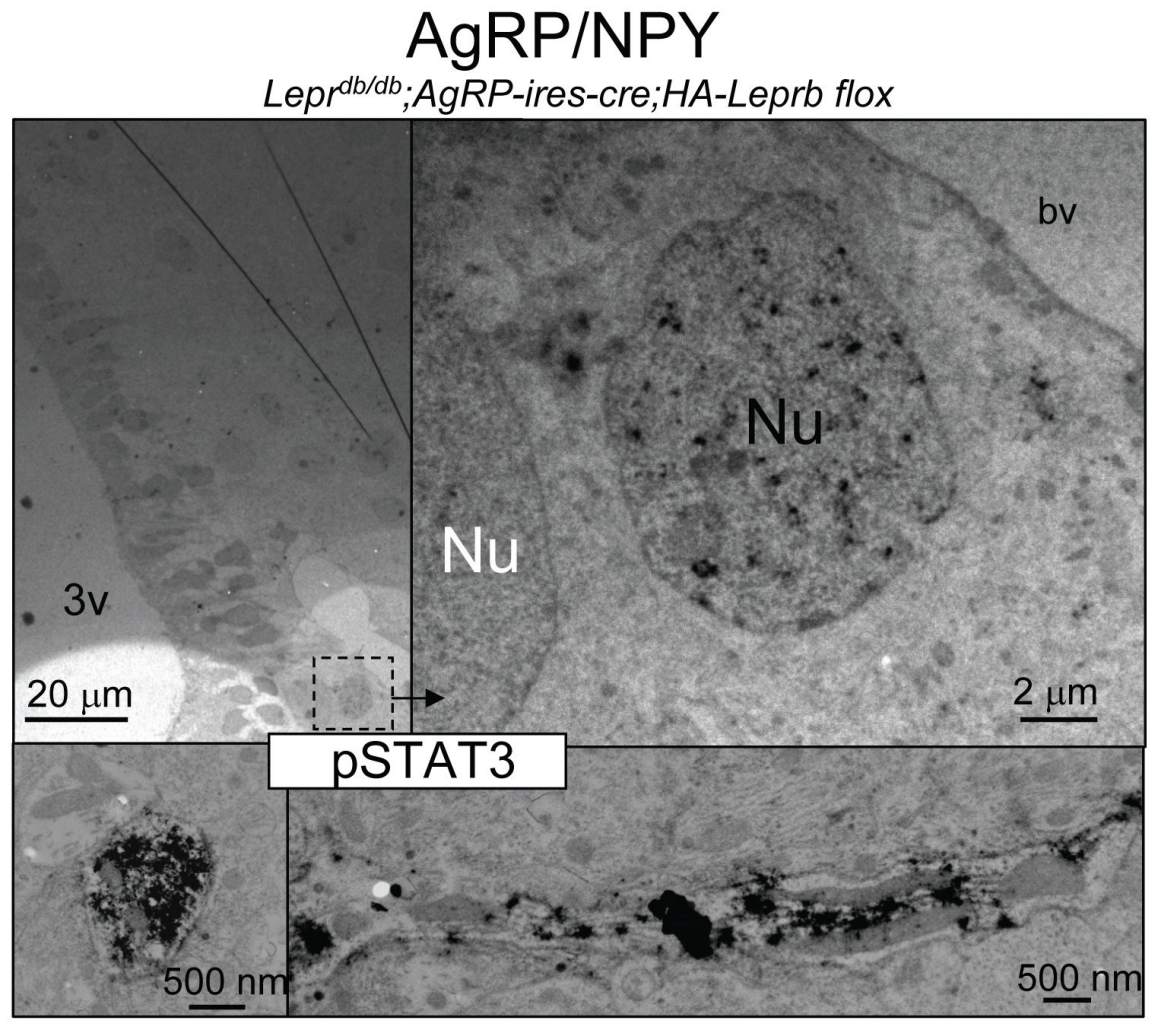
Activation of STAT3 phosphorylation by leptin in dendrites of hypothalamic AgRP neurons by immuno-electron microscopy (EM). Immuno-EM for pSTAT3 (DAB) in a leptin-treated (4 mg/kg ip; 10 min) *Lepr* ^*db/db*^;*Agrp-ires-cre;HA-LepRb flox* mouse. Left Top: Overview of medial-arcuate region. Top Right: Magnification of box from left. pSTAT3 IR in a neuronal nucleus (“Nu” in black text) and a non-pSTAT3 IR nucleus is labeled with “Nu” (white text). Bottom Left: Cross-section of a pSTAT3 IR dendrite (EM @ 30,000x). Bottom Right: A pSTAT3 IR dendrite in the horizontal plane (EM @ 1,200x). bv: blood vessel.

Figure 8 shows pSTAT3 immuno-EM microphotographs at different levels of magnification from the medial Arc of a leptin-treated *Lepr* ^*db/db*^;*Pomc-cre;HA-Leprb flox* mouse. In the left image, pSTAT3 IR neuronal nuclei (black arrow) and non-pSTAT3 IR nuclei (white arrow) can be seen. At higher magnifications, pSTAT3 IR dendritic shaft structures are identified, including dendrites in the photographic plane (top right). An example of a cross-section of a large dendrite with an array of microtubules (MT) and one mitochondrion (M) is presented at bottom right. In several brain sections from several grids, we identified random pSTAT3 IR structures and counted a majority of neuronal nuclei (N=49) and dendrites (N=70), and no axonal fibers or presynaptic structures (N=0). At this time-point after leptin treatment, immunostaining was rarely seen in the cytoplasm (soma) of neurons with pSTAT3 IR nuclei. Similarly, shown in Figure 9 are different direct magnification levels of brain sections from a leptin-treated *Lepr* ^*db/db*^;*AgRP-ires-cre;HA-Leprb flox* mouse. Many pSTAT3 IR neuronal nuclei and dendritic structures are identified. Analysis of randomly selected pSTAT3 IR structures identified neuronal nuclei (N=16), dendrites (N=358) and axons (N=1). Because these above studies were done in genetically modified and obese *Lepr* ^*db/db*^ mice, we also investigated pSTAT3 IR cellular structures in the medial arcuate nucleus of leptin-treated wild type C57BL/6J mice (Figure S8). Of randomly selected pSTAT3 IR cellular structures, we counted neuronal nuclei (N=16) and dendrites (N=79), and found no evidence of pSTAT3 IR in axonal structures (N=0). We did not find pSTAT3 IR in macroglia, including in astrocytes, microglia or ependymal cells within the arcuate of these wt mice suggesting that these cell types may not express LepRb. Altogether, these EM studies show somato-dendritic localization of leptin-stimulated pSTAT3 both in random arcuate neurons of wild type mice, and in POMC and AgRP neurons of transgenic *Lepr* ^*db/db*^;*Pomc-cre;HA-Leprb flox* and *Lepr* ^*db/db*^;*AgRP-ires-cre;HA-Leprb flox* mice, consistent with the somato-dendritic expression pattern of LepRb shown above.

### Leptin activates STAT3 in proximity to post-synaptic structures on dendritic shafts from POMC and AgRP/NPY neurons

**Figure 10:**
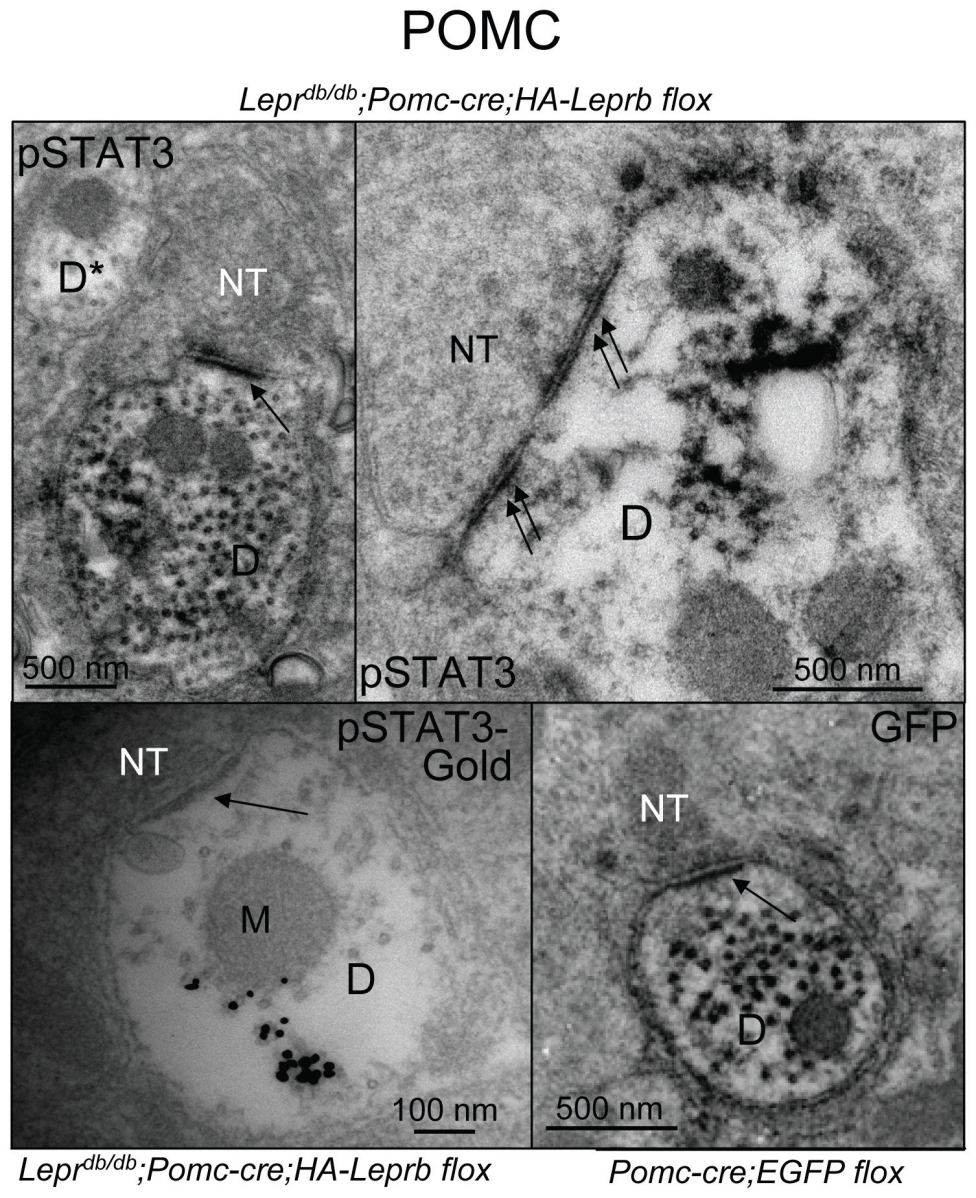
Leptin activates STAT3 signaling in proximity to post-synaptic structures on dendritic shafts from POMC neurons. Top Left: Immuno-EM for pSTAT3 (DAB) from a leptin-treated (4 mg/kg ip; 10 min) *Lepr^db/db^*;*POMC-cre;HA-LepRb* *flox* mouse. Arrow indicates an asymmetrical synapse on a cross section of a pSTAT3 IR dendritic shaft (D). The presynaptic nerve terminal is labeled (NT). A non-pSTAT3 IR dendrite is also marked (D*). Top Right: Example of a pSTAT3 IR dendritic shaft with a symmetrical synapse (double arrows). Bottom Left: Immuno-Gold EM for pSTAT3 from a leptin-treated (4 mg/kg ip; 10 min) *Lepr^db/db^*;*POMC-cre;HA-LepRb* *flox* mouse. Arrow indicates PSD of an asymmetrical synapse. Bottom Right: Immuno-EM for GFP (DAB) from a *Pomc-cre;EGFP* *flox* mouse. Arrow indicates an asymmetrical synapse on a cross section of a GFP IR (POMC) dendritic shaft. PSD: post-synaptic density.

**Figure 11:**
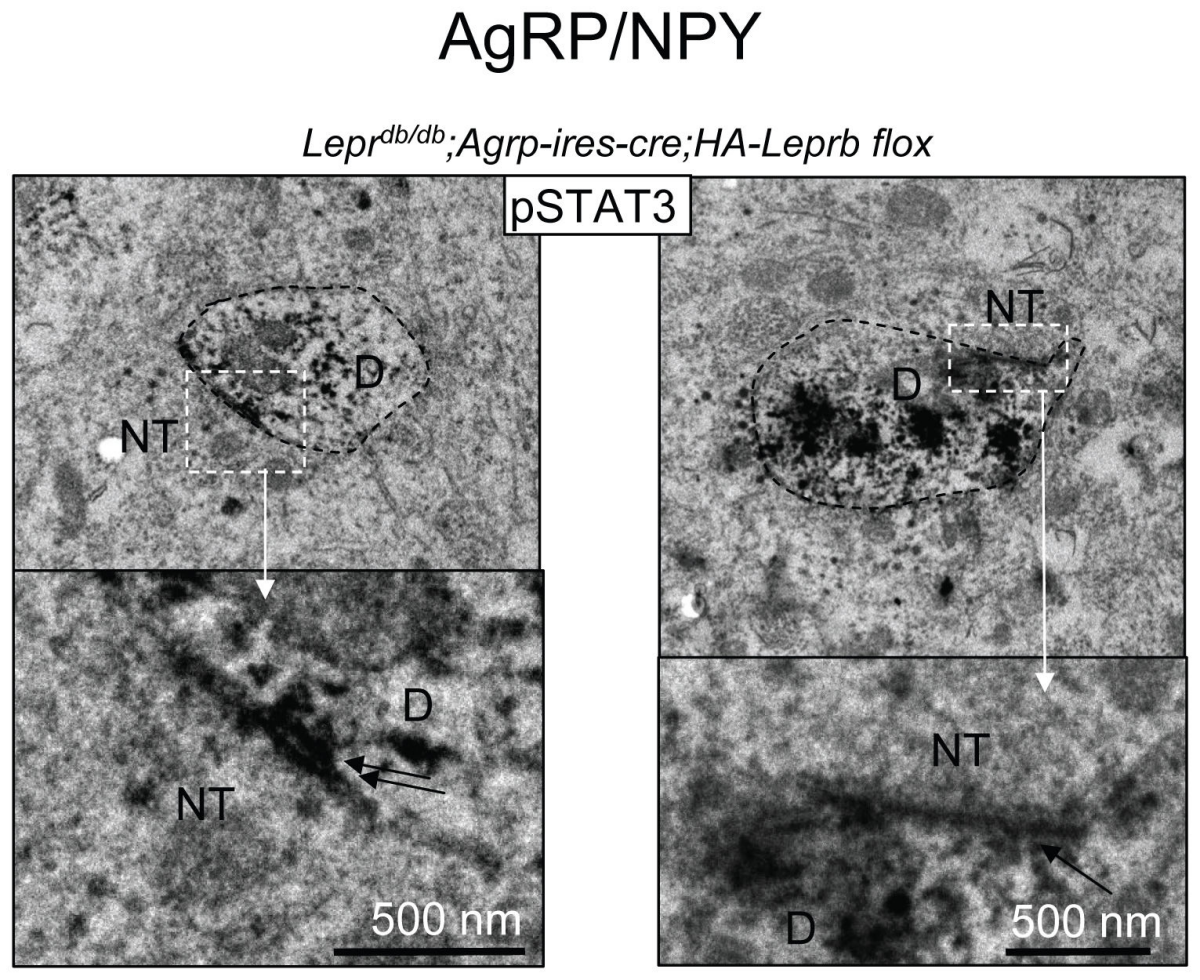
Leptin activates STAT3 signaling in proximity to post-synaptic structures on dendritic shafts from AgRP neurons. Left Side: Immuno-EM for pSTAT3 (DAB) from a leptin-treated (4 mg/kg ip; 10 min) *Lepr^db/db^*;*AgRP-cre;HA-LepRb* *flox* mouse. The dendrite (D) is marked by black stippled line. Bottom: Magnification of symmetrical synaptic structure from stippled box above. Double arrows indicate the PSD. Right Side: Immuno-EM for pSTAT3 (DAB) in cross section of dendritic shaft from a leptin-treated (4 mg/kg ip; 10 min) *Lepr^db/db^*;*AgRP-cre;HA-LepRb* *flox* mouse. The dendrite (D) is marked by a black stippled line. Bottom: Magnification of the asymmetrical synapse from above. Single black arrow indicates the PSD. D: dendrite (pSTAT3 IR positive); D*: dendrite (pSTAT3 IR negative); M: mitochondrion; NT: presynaptic nerve terminal.

To further explore the sub-cellular localization of leptin receptor signaling within dendrites, we examined possible leptin-induced STAT3 phosphorylation within dendritic spines and in proximity to post-synaptic structures on dendritic shafts of POMC and AgRP neurons. In randomly selected pSTAT3 IR (POMC) dendritic structures in brains of *Lepr* ^*db/db*^;*Pomc-cre;HA-Leprb flox* mice we did not find direct evidence of spines by EM. This is consistent with earlier reports by Hentges et al. [59] and Liu et al. [16] showing a low number of dendritic spines on POMC neurons. By CLSM, which allows rapid analysis of much larger fields and longer fiber distances, we did however observe pSTAT3 IR within rare spine-like structures on POMC dendrites (Figure S9). By EM, phospho-STAT3 IR was detected in dendritic shafts in close proximity to both asymmetrical and symmetrical synapses (Figure 10). The pSTAT3 IR did not appear to concentrate directly within the post-synaptic density (PSD) itself, but was mostly associated with microtubules. Of note, pSTAT3 IR was not detected within mitochondria. Because it can be difficult to clearly differentiate the black DAB deposits from the electron-dense post-synaptic density, we employed pSTAT3 immuno-Gold EM. As shown in Figure 10, gold particles were indeed targeted to microtubules and not directly to the PSD itself. We also demonstrated presence of asymmetrical shaft synapses on POMC dendrites in brains of lean *Pomc-cre;EGFP lox* mice by immuno-GFP EM (Figure 10). In AgRP dendrites, we similarly detected leptin-induced pSTAT3 IR in proximity to both symmetrical and asymmetrical synapses on dendritic shafts (Figure 11). However in contrast to the reported high-density of dendritic spines on AgRP neurons relative to POMC neurons [16], we did not find evidence of spines in pSTAT3 IR dendritic structures in leptin-treated *Lepr* ^*db/db*^;*AgRP-ires-cre;HA-Leprb flox* mice.

### 3D-reconstruction of hypothalamic POMC and AgRP/NPY dendritic segments

To further examine LepRb-dependent STAT3 phosphorylation in POMC and AgRP dendrites, we performed serial-EM 3D-reconstruction of pSTAT3 IR dendritic segments from leptin-treated *Lepr* ^*db/db*^;*Pomc-cre;HA-Leprb flox* and *Lepr* ^*db/db*^;*AgRP-ires-cre;HA-Leprb flox* mice. 

**Figure 12:**
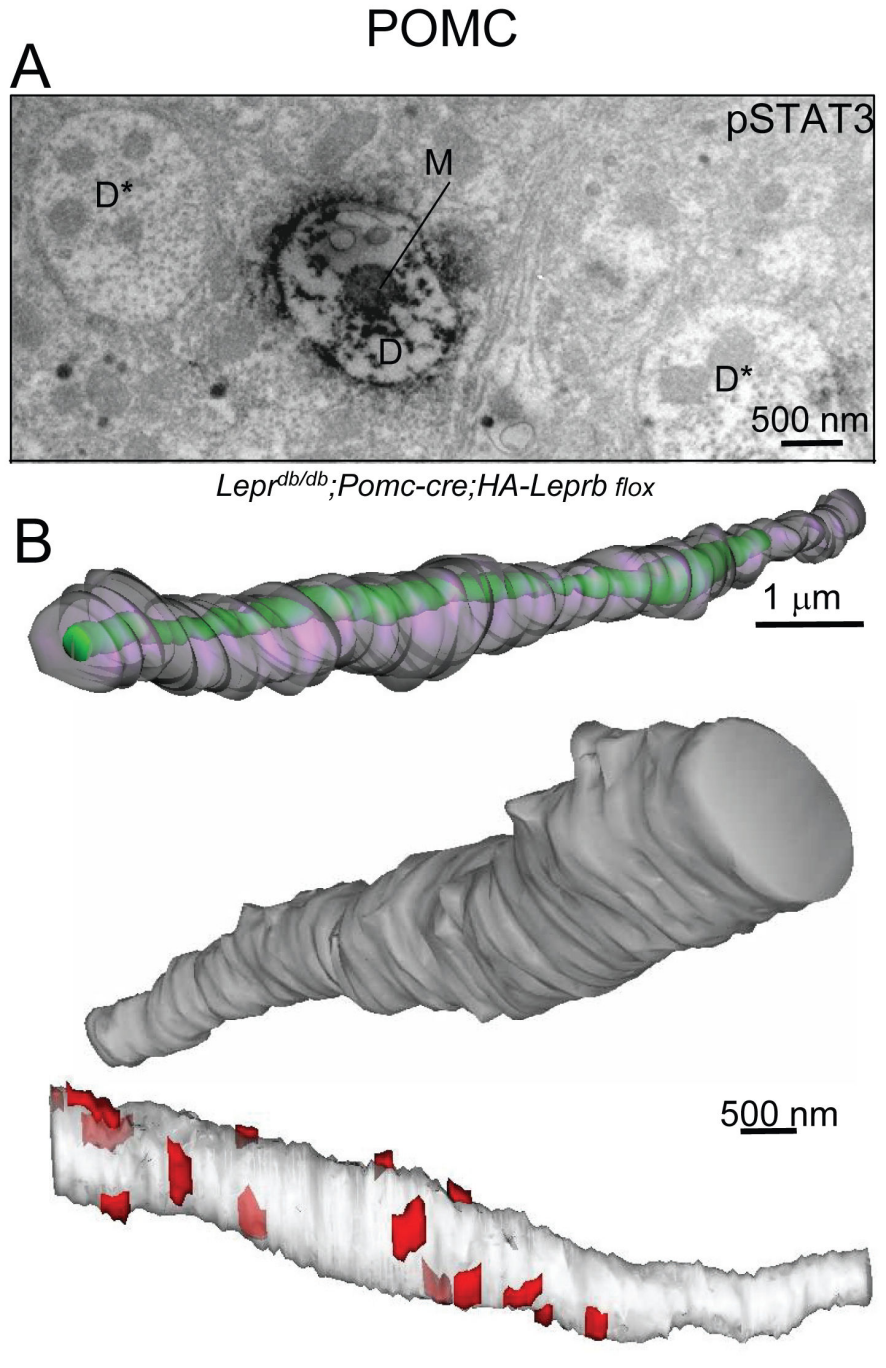
3D-reconstruction of a leptin-responsive hypothalamic POMC dendritic segment. **A**. Immuno-EM for pSTAT3 (DAB) in a leptin-treated *Lepr* ^*db/db*^;*Pomc-cre;HA-LepRb* *flox* mouse. Cross-section of a pSTAT3 IR dendritic shaft with a single mitochondrion are shown. Two non-pSTAT3 IR dendrites also marked (D*). **B**. Serial-EM 3D-reconstruction of the POMC dendritic shaft of the pSTAT3 IR dendrite in Figure A. Top: Shown is a partially transparent presentation (mitochondrion in green and the dendritic surface in gray). Middle: Non-transparent surface representation of same POMC dendritic shaft segment (after rotation). Bottom: Shown is a partially transparent representation with shaft synapses depicted in red (N=15). Total length of segment is ~8.0 µm (reconstructed from 160 sections - each ~50 nm thick). D: dendrite; M: mitochondrion.

Figure 12A shows a cross-section of a large pSTAT3 IR dendritic shaft in a brain section from a leptin-treated *Lepr* ^*db/db*^;*Pomc-cre;HA-Leprb flox* mouse. One mitochondrion is present. The dendrite was followed through 160 adjacent 50 nm thick EM sections and reconstructed in 3-dimensions using the Reconstruct software. Figure 12B shows a semi-transparent representation of the ~8.0 µm long segment with the single intracellular mitochondrion (green). This segment did not contain any spine structures consistent with the above analyses of random pSTAT3 IR dendritic structures and the earlier report by *Liu et al* [16].. A different dendritic segment (11 µm long) was also reconstructed and similarly did not show evidence of spines (not shown). Figure 12B (middle) shows a non-transparent surface presentation. Finally, we identified shaft synapses on the same segment. A total of 15 synaptic structures were found along the segment and are presented in red on a semi-transparent presentation in Figure 12 (bottom).

**Figure 13:**
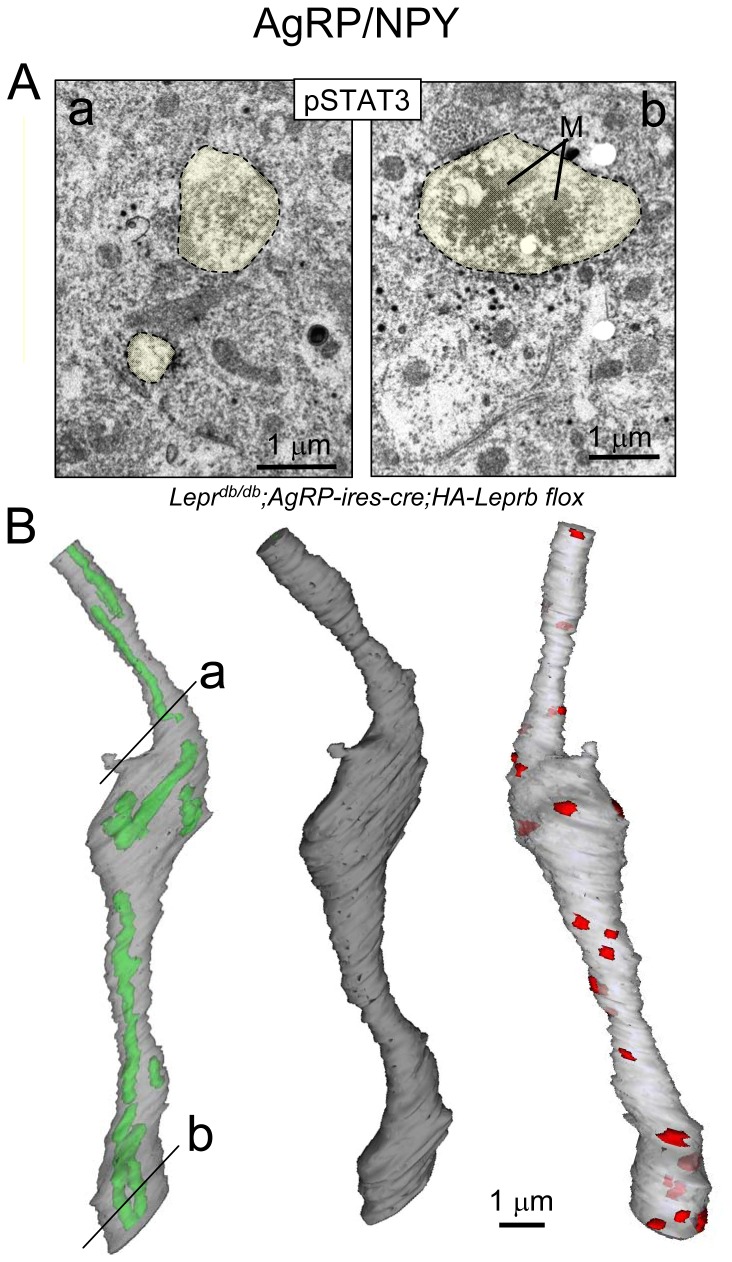
3D-reconstruction of a leptin-responsive hypothalamic AgRP dendritic segment. **A**. Immuno-EM for pSTAT3 (DAB) in a leptin-treated *Lepr* ^*db/db*^;*AgRP-ires-cre;HA-LepRb* *flox* mouse. Left (a): Cross sections of the dendritic shaft and the spine-like structure are outlined by stippled lines and by yellow transparent color. Right (b): Shown is a cross section of same dendritic shaft, but at a different level. Two mitochondria are labeled (M). **B**: Left: 3D-reconstruction of the dendritic segment. The location of the two EM microphotographs from A are marked (a and b). Shown is a partially transparent representation with intracellular mitochondria in green and the dendritic surface in gray. Middle: Non-transparent surface representation. Bottom: Shown is a partially transparent representation with shaft synapses depicted in red (N=20). Total length is ~12.5 µm (250 sections – each ~50 nm thick).

We similarly reconstructed a pSTAT3 IR dendritic shaft-segment from a leptin-treated *Lepr* ^*db/db*^;*Pomc-cre;HA-Leprb flox* mouse. Figure 13A shows microphotographs of two cross sections of the pSTAT3 IR dendrite at different levels (*a* and *b*). The 3D-reconstruction of the ~12.5 μm long segment including its multiple mitochondria (green) and one spine-like structure is presented in Figure 13B (left). A different AgRP dendrite segment (3.2 μm long) was also examined and did not exhibit any spines (not shown). A non-transparent surface representation is shown in Figure 13B (middle). This AgRP dendritic segment had 20 identifiable synapses (Figure 13B, right).

Combined, these ultrastructural data show leptin-dependent STAT3 phosphorylation within POMC and AgRP/NPY dendrites in close proximity to both symmetrical and asymmetrical shaft synapses. 

## Discussion

The principal finding of these studies is that the long signaling form of the leptin receptor, LepRb, exhibits a somato-dendritic expression pattern in hypothalamic arcuate POMC and AgRP/NPY neurons. In addition, we show evidence of leptin-dependent LepRb signaling in close proximity to synaptic structures on dendritic shafts. Combined, these results suggest that leptin has dendritic actions that may involve modulation of synaptic function and be important for mediating leptin’s *in vivo* metabolic actions.

The dendritic localization of LepRb is supported by the following findings: 1) Leptin-dependent STAT3 phosphorylation is found in relatively short neuronal fibers that do not extend significantly beyond each brain nucleus; 2) HA-LepRb is directly localized to proximal and distal dendritic fibers of POMC neurons; and 3) ultrastructual studies show STAT3 activation by leptin in dendrites in POMC and AgRP neurons. The lack of axonal LepRb expression and signaling is attributed to: 1) HA-LepRb immuno-reactivity is not found in POMC IR or β-Endorphin IR fibers; 2) leptin-activated pSTAT3 is not detected in α-MSH processes; 3) leptin-stimulated pSTAT3 is lacking in known axonal terminal beds of POMC and AgRP neurons, such as the PVH; 4) EM analyses did not show evidence of pSTAT3 in axon fibers of POMC and AgRP/NPY neurons in transgenic mice selectively expressing LepRb only in those neurons; and 5) EM analyses show negative results for pSTAT3 in axon fibers within the arcuate nucleus of wild-type animals. Despite this evidence, we cannot rule out the possibility that low levels of LepRb are present in axons and/or that STAT3 is activated in axons at either longer or shorter time points following *in vivo* leptin administration.

Somato-dendritic expression and signaling by LepRb is consistent with a recent study reporting effects of leptin on translation of brain-derived neurotrophic factor (BDNF) mRNA in dendritic fibers of VMH neurons [60]. In addition, a similar localization pattern has been reported for CNTF-activated pSTAT3 in cranial and spinal motor neurons [61]. Also, ultrastructural immuno-EM analyses in rodent brains of non-cytokine receptors such as somatostatin receptor subtypes (sst1) [62], the mu-opioid receptor (MOR) [63] and the corticotrophin-releasing factor receptor (CRFr) have shown a predominant somato-dendritic sub-cellular compartmentalization [64]. 

 Relative to the soma, dendrites contain the vast majority of synapses on a given neuron. Our ultrastructural experiments showing leptin-dependent STAT3 phosphorylation in proximity to symmetrical and asymmetrical synapses, suggest that LepRb signaling might influence post-synaptic responses to GABA and glutamate. Indeed, electrophysiological slice studies of hippocampal neurons show that leptin can enhance the amplitude of excitatory post synaptic currents (EPSCs) via N-methyl-D-aspartate receptors (NMDAR) and α-amino-3-hydroxy-5-methyl-4-isoxazolepropionic acid receptors (AMPAR)[35,65]. Leptin did not modulate the paired pulse ratio (PPr), further indicating involvement of a postsynaptic mechanism rather than affecting glutamate release (pre-synaptic action). In addition, leptin facilitates NMDA-induced Ca^2+^ influx in dissociated hippocampal and cerebellar neuronal cultures and in *Xenopus* oocytes transfected with LepRb- and NMDAR-encoding plasmids [35,36]. Combined, these findings are consistent with dendritic expression of LepRb. 

An early study of leptin’s electrophysiological effects suggested that LepRb is localized to pre-synaptic axon-terminal structures of hypothalamic NPY (AgRP) neurons serving to influence GABA release onto POMC neurons [12]. However, this conclusion was based on measurements of mini inhibitory-postsynaptic currents (mIPSCs) in only 3 POMC neurons. In addition, the effect of leptin on POMC IPSC frequency was slow (~12-15 minutes) leaving a pre-synaptic somato-dendritic based mechanism feasible. For example, LepRb localized to the soma of NPY neurons might influence transfer of GABA-related substances from the soma to nerve terminals via the microtubule transport system [66-68]. Indeed, fast anterograde axonal transport can occur at rate between 140-280 µm/minute, and conveyed in this system is a variety of proteins, synaptic vesicles and enzymes associated with neurotransmitter generation [66-68]. Alternatively, with the delayed leptin time-response in mind [12], a post-synaptic mechanism involving restructuring of the dendritic receptive zone (synaptogenesis) via LepRb expressed in POMC dendrites might also be possible [69-71]. Future electrophysiological studies involving mice with specific deletion of LepRb from POMC or NPY/AgRP/GABA neurons might differentiate between a pre-synaptic versus a post-synaptic mechanism for leptin to modulate PSCs in this neurocircuitry. 

A more recent study reported inhibition of glutamate release by leptin onto neurons in the ventral tegmental area (VTA) and similarly to Cowley et al. [12], also showed a schematic figure depicting localization of LepRb to the nerve terminal of the presynaptic neuron [37]. This conclusion was based on measurements in brain slices showing a reduction in the probability of glutamate release onto VTA neurons. In addition, the depressive effects of leptin on AMPAR and NMDAR mini excitatory-postsynaptic currents (mEPSCs) affected frequency, but not amplitude. Such results are typically consistent with a pre-synaptic mechanism of LepRb action as proposed in the paper, and it is therefore possible that LepRb is localized differently in POMC and AgRP/NPY neurons versus other types of LepRb neurons (e.g. the glutamatergic neurons that synapse onto VTA neurons). On the other hand, the studies by Thompson et al. [37] cannot entirely exclude the possibility that the pre-synaptic actions of leptin are mediated by LepRb localized to the somata (and/or dendrites) of the glutamatergic neurons. As also discussed above, the relatively slow time-course for the effect on mEPSCs (15-18 minutes after leptin) and PPr (30 minutes after leptin) could allow enough time for leptin-action on the soma to influence transport of transmitter-related substances to nerve terminals. Indeed, we show evidence of LepRb membrane localization in the soma of arcuate POMC neurons (although these are likely not the glutamatergic neurons innervating the VTA). Future studies will be needed to decisively determine whether the above reported pre-synaptic actions on VTA neurons occur via LepRb signaling in the soma/dendrites or within axon terminals of the pre-synaptic neurons.

We also show evidence that STAT3, a transcription factor, is activated in distal dendrites. However its function in this neuronal compartment is unknown. One possibility is that STAT3 is eventually transported to the soma/nucleus to influence gene expression. Consistent with this possibility we found that pSTAT3 is associated with microtubules, the major transportation machinery in dendrites [72]. Earlier studies in non-neuronal cell models have reported interaction between STAT3 and microtubule-associated proteins, and effects of STAT3 on microtubule stabilization [73,74]. The LepRb-STAT3 pathway might thus affect dendritic growth/branching and/or neuronal migration and/or synaptic plasticity [58,70,75-77]. Alternatively, STAT3 may serve a novel function in dendrites that has yet to be determined. Of additional interest for further studies is the possibility of a dendritic localization-signal-sequence within LepRb, as has been reported in other proteins with somato-dendritic compartment localization [78-82].

Consistent with the recent studies by Liu et al. [16], we found only few spines on POMC dendrites. However, in contrast to those studies by Liu et al., we found that dendrites of AgRP/NPY neurons also have few spine structures. One possible reason for this discrepancy is that our EM studies are limited to investigations of a low number of dendritic segments that might not be representative. It is also possible that AgRP dendrites have lost spine expression due to lack of LepRb expression in neurons (except AgRP neurons) in the *Leprb* ^*db/db*^ *; Agrp-ires-cre; HA-Leprb flox* mice, or indirectly because of the obesity or other abnormalities of these animals. Alternatively, STAT3 may activated by leptin in regions of AgRP/NPY/GABA dendrites that have a low density of spines. Our 3D-reconstructions further indicate that the number of shaft synapses may greatly out-number spine synapses on both POMC and AgRP/NPY dendrites. We also show evidence that many of the shaft synapses can be categorized as either symmetrical or asymmetrical, which typically represents GABAergic and glutamatergic synapses, respectively [83,84]. The GABAergic and glutamatergic synaptic input measured on POMC and AgRP/NPY/GABA neurons may therefore primarily involve dendritic shaft synapses rather than dendritic spine synapses [12,16,85]. 

 Further implications of our finding of dendritic LepRb localization include the possibility that leptin-resistant obesity may be caused, at least in part, by altered LepRb expression or signaling in this neuronal compartment. In addition, it will be important to determine if LepRb itself is localized and signals directly within the post-synaptic density to affect synaptic activity.

## Supporting Information

Figure S1 **Leptin activates STAT3 phosphorylation in neuronal fiber processes within the NTS of C57BL/6J mice.** Shown are light microscopy (LM) images of phospho-STAT3IR (DAB) in coronal brain sections of the hindbrain from 8 weeks old wild type C57BL/6J male mice. Animals were given leptin (5 mg/kg, ip) and sacrificed after 30 minutes. Top: pSTAT3 IR is found within the NTS at the level of the area postrema (AP). The bottom image shows high magnification of stippled box from top. Arrows identify some of many pSTAT3 IR neuronal processes. XII: Hypoglossal nerve; NTS: nucleus of the solitary tract; DMX: dorsal motor nucleus of the vagus nerve; cc: central canal, AP: area postrema.
(TIF)

Figure S2 **Leptin does not induce STAT3 phosphorylation within the PVH, a major axonal target zone of leptin-responsive POMC neurons.** Left: pSTAT3 IR is present in the RCH/Arc region of the anterior hypothalamus, but importantly, not in the PVH of leptin-treated (5 mg/kg i.p., 30 minutes) C57BL/6J mice. Bottom: High-magnification microphotograph demonstrating lack of pSTAT3 IR fibers (and nuclei) in the PVH. Right: Many POMC IR nuclei are found in the RCH/Arc. Bottom: Dense networks of neuronal (axonal) POMC fibers are observed in PVH. RCH: retrochiasmatic area; Arc: arcuate; 3v: 3^rd^ ventricle; PVH: paraventricular hypothalamic nucleus.
(TIF)

Figure S3 **Leptin activates STAT3 phosphorylation in neuronal fiber processes in hypothalamic nuclei of Sprague Dawley rats.** Left: Shown are light microscopy (LM) images of phospho-STAT3 IR (DAB) in coronal brain sections of the mediobasal hypothalamus from Sprague Dawley rats. Rats were given leptin (5 mg/kg, ip) for 45 minutes. . Right: High-magnification microphotographs of boxes in left column. Robust pSTAT3 IR is found in fibers within the VMH and PMN.
(TIF)

Figure S4 **Expression of HA-tagged LepRb in hypothalamic POMC neurons.** **A**. CLSM of POMC neurons (red (POMC-polypeptide IR)) and HA-LepRb (green (HA IR)) in a hypothalamic brain section from a *Pomc-cre;HA-Leprb* *flox* mouse (top row) and a negative control section from a *HA-Leprb* *flox* mouse (bottom row). Some non-specific HA IR (green) is observed along the lining of the 3rd ventricle and at the base of the Arc in the control section. Shown are single confocal planes. **B**. Top row: Example of a POMC soma (red) co-expressing HA-LepRb (green) in a *Pomc-cre;HA-Leprb* *flox* mouse. DAPI fluorescence (blue) identifies the nucleus. Bottom row: Example of a POMC neuron that does not express HA-LepRb in a *HA-Leprb* *flox* control mouse. Shown are single confocal planes.
(TIF)

Figure S5 **Localization of leptin receptors and activation of STAT3 in neuronal fibers of transfected primary neurons.** **A**. Primary hypothalamic neurons were co-transfected with plasmids encoding HA-tagged LepRb and GFP, and subjected to immunocytochemistry (ICC) for HA (green) and GFP (red). Leptin receptors are expressed in the soma and in fibers. Shown are collapsed confocal Z-stack sections **B**. As in A., neurons were transfected with plasmids encoding HA-LepRb. At DIV 12, cells were treated 100 nM leptin for 20 min and fixed. Slides were then subjected to ICC for pSTAT3 (green) and PSD95, a dendritic protein marker (red). DAPI (blue) was included to label nuclei. Top: Two neurons exhibit pSTAT3 IR in the soma and fibers. Bottom: Enlargement of box in top image showing punctate pSTAT3 staining in PSD95 positive fibers. Shown are single confocal planes. DIV: days in vitro.
(TIF)

Figure S6 **Lepr^db/db^** ;***Pomc-cre;HA-LepRbflox* mice express pSTAT3 in neuronal processes of POMC neurons**.
Top Left: LM shows lack of pSTAT3 IR (DAB) in the mediobasal hypothalamus of a leptin-treated (5 mg/kg, ip, 20 min) obese *Lepr* ^*db/db*^ control mouse. Bottom Left: pSTAT3 IR in the arcuate (ARC), but not the VMH or LHA of obese *Lepr^db/db^*;*Pomc-cre;HA-LepRb* *flox* mice consistent with the targeting of HA-LepRb to POMC neurons. Insert: For comparison, the anatomical localization of POMC neurons is shown by *in* *situ* hybridization for *Pomc* mRNA. Right: Enlargement of box. Many positive pSTAT3 fibers are shown (arrows). 3v: 3^rd^ ventricle; Arcuate: hypothalamic arcuate nucleus; VMH: ventromedial hypothalamic nucleus; LHA: lateral hypothalamic area.
(TIF)

Figure S7 **Lepr^db/db^** ;***Pomc-cre;HA-LepRbflox* mice express pSTAT3 in neuronal processes of AgRP neurons**.
Left: LM shows pSTAT3 IR (DAB) in the mediobasal hypothalamus of a leptin-treated (5 mg/kg, ip, 20 min) obese *Lepr^db/db^*;*Pomc-cre;HA-LepRb* *flox* mouse. pSTAT3 IR is found in the medial arcuate (ARC), but not in the VMH or LHA, consistent with targeting of HA-LepRb expression to AgRP neurons. Insert: For comparison, the anatomical localization of AgRP/NPY neurons is shown by *in* *situ* hybridization for *Npy* mRNA. Right: Enlargement of box. Several pSTAT3 IR fibers are visible (arrows). 3v: 3^rd^ ventricle; VMH: ventromedial hypothalamic nucleus; LHA: lateral hypothalamic area.
(TIF)

Figure S8 **Activation of STAT3 phosphorylation by leptin in dendrites of arcuate hypothalamic neurons of wild type C57BL/6J mice.** Immuno-EM for pSTAT3 (DAB) in the arcuate hypothalamic nucleus of a leptin-treated (4 mg/kg, ip, 15 min) wild type C57BL/6J mouse. Top: A pSTAT3 IR neuronal nucleus is labeled with “Nu” (black text) and several pSTAT3 IR dendrites are indicated with black arrows. A pSTAT3 IR negative neuronal nucleus is labeled “Nu” in white text. Insert: A cross sections of a pSTAT3 IR dendritic shaft. Bottom: pSTAT3 IR dendrite in the photographic plane. Nu; nucleus; D: dendrite; bv: blood microvessel.
(TIF)

Figure S9 **Activation of STAT3 phosphorylation by leptin in dendritic spines of POMC neurons.** A *Pomc-EGFP* mouse was given leptin (5 mg/kg, ip, 30 min). IHC and CLSM was applied to visualize pSTAT3 IR (red) and EGFP-epifluorescence (green) in a brain section of the arcuate nucleus of the hypothalamus. All images are single confocal planes. The arrow depicts a dendritic spine-like structure.
(TIF)

## References

[1] Ahima RS, Antwi DA (2008) Brain regulation of appetite and satiety. Endocrinol Metab Clin North Am 37: 811-823. doi:10.1016/j.ecl.2008.08.005. PubMed: 19026933. 19026933 PMC2710609

[2] Robertson SA, Leinninger GM, Myers MG Jr (2008) Molecular and neural mediators of leptin action. Physiol Behav 94: 637-642. doi:10.1016/j.physbeh.2008.04.005. PubMed: 18501391. 18501391 PMC2516921

[3] Friedman JM (2009) Leptin at 14 y of age: an ongoing story. Am J Clin Nutr 89: 973S-979S. doi:10.3945/ajcn.2008.26788B. PubMed: 19190071. 19190071 PMC2667654

[4] Coppari R, Bjørbaek C (2012) Leptin revisited: its mechanism of action and potential for treating diabetes. Nat Rev Drug Discov 11: 692-708. doi:10.1038/nrd3757. PubMed: 22935803. 22935803 PMC4019022

[5] Hahn TM, Breininger JF, Baskin DG, Schwartz MW (1998) Coexpression of Agrp and NPY in fasting-activated hypothalamic neurons. Nat Neurosci 1: 271-272. doi:10.1038/1082. PubMed: 10195157. 10195157

[6] Wilson BD, Bagnol D, Kaelin CB, Ollmann MM, Gantz I et al. (1999) Physiological and anatomical circuitry between Agouti-related protein and leptin signaling. Endocrinology 140: 2387-2397. doi:10.1210/en.140.5.2387. PubMed: 10218993. 10218993

[7] Mercer AJ, Hentges ST, Meshul CK, Low MJ (2013) Unraveling the central proopiomelanocortin neural circuits. Front Neurosci 7: 19 PubMed: 23440036. 23440036 10.3389/fnins.2013.00019 PMC3579188

[8] Aponte Y, Atasoy D, Sternson SM (2011) AGRP neurons are sufficient to orchestrate feeding behavior rapidly and without training. Nat Neurosci 14: 351-355. doi:10.1038/nn.2739. PubMed: 21209617. 21209617 PMC3049940

[9] Krashes MJ, Koda S, Ye C, Rogan SC, Adams AC et al. (2011) Rapid, reversible activation of AgRP neurons drives feeding behavior in mice. J Clin Invest 121: 1424-1428. doi:10.1172/JCI46229. PubMed: 21364278. 21364278 PMC3069789

[10] Yang Y, Atasoy D, Su HH, Sternson SM (2011) Hunger states switch a flip-flop memory circuit via a synaptic AMPK-dependent positive feedback loop. Cell 146: 992-1003. doi:10.1016/j.cell.2011.07.039. PubMed: 21925320. 21925320 PMC3209501

[11] Elias CF, Aschkenasi C, Lee C, Kelly J, Ahima RS, Bjorbaek C et al. (1999) Leptin differentially regulates NPY and POMC neurons projecting to the lateral hypothalamic area. Neuron 23: 775-786. doi:10.1016/S0896-6273(01)80035-0. PubMed: 10482243. 10482243

[12] Cowley MA, Smart JL, Rubinstein M, Cerdán MG, Diano S et al. (2001) Leptin activates anorexigenic POMC neurons through a neural network in the arcuate nucleus. Nature 411: 480-484. doi:10.1038/35078085. PubMed: 11373681. 11373681

[13] van den Top M, Lee K, Whyment AD, Blanks AM, Spanswick D (2004) Orexigen-sensitive NPY/AgRP pacemaker neurons in the hypothalamic arcuate nucleus. Nat Neurosci 7: 493-494. doi:10.1038/nn1226. PubMed: 15097991. 15097991

[14] Huang H, Lee SH, Ye C, Lima IS, Oh BC et al. (2013) ROCK1 in AgRP neurons regulates energy expenditure and locomotor activity in male mice. Endocrinology [Epub ahead of print]. PubMed: 23885017. 10.1210/en.2013-1343 PMC3776869 23885017

[15] Takahashi KA, Cone RD (2005) Fasting induces a large, leptin-dependent increase in the intrinsic action potential frequency of orexigenic arcuate nucleus neuropeptide Y/Agouti-related protein neurons. Endocrinology 146: 1043-1047. PubMed: 15591135. 15591135 10.1210/en.2004-1397

[16] Liu T, Kong D, Shah BP, Ye C, Koda S et al. (2012) Fasting Activation of AgRP Neurons Requires NMDA Receptors and Involves Spinogenesis and Increased Excitatory Tone. Neuron 73: 511-522. doi:10.1016/j.neuron.2011.11.027. PubMed: 22325203. 22325203 PMC3278709

[17] Balthasar N, Coppari R, McMinn J, Liu SM, Lee CE et al. (2004) Leptin receptor signaling in POMC neurons is required for normal body weight homeostasis. Neuron 42: 983-991. doi:10.1016/j.neuron.2004.06.004. PubMed: 15207242. 15207242

[18] van de Wall E, Leshan R, Xu AW, Balthasar N, Coppari R et al. (2008) Collective and individual functions of leptin receptor modulated neurons controlling metabolism and ingestion. Endocrinology 149: 1773-1785. doi:10.1210/en.2007-1132. PubMed: 18162515. 18162515 PMC2276717

[19] Münzberg H, Flier JS, Bjørbaek C (2004) Region-Specific leptin resistance within the hypothalamus of diet-induced obese mice. Endocrinology 145: 4880-4889. doi:10.1210/en.2004-0726. PubMed: 15271881. 15271881

[20] Enriori PJ, Evans AE, Sinnayah P, Jobst EE, Tonelli-Lemos L et al. (2007) Diet-induced obesity causes severe but reversible leptin resistance in arcuate melanocortin neurons. Cell Metab 5: 181-194. doi:10.1016/j.cmet.2007.02.004. PubMed: 17339026. 17339026

[21] Gamber KM, Huo L, Ha S, Hairston JE, Greeley S, Bjorbaek C (2012) Over-expression of leptin receptors in POMC neurons promotes development of diet-induced obesity. PLOS ONE 7: e30485. doi:10.1371/journal.pone.0030485. PubMed: 22276206. 22276206 PMC3262822

[22] Thompson JL, Borgland SL (2013) Presynaptic leptin action suppresses excitatory synaptic transmission onto ventral tegmental area dopamine neurons. Biol Psychiatry 73: 860-868. doi:10.1016/j.biopsych.2012.10.026. PubMed: 23305991. 23305991

[23] Lee GH, Proenca R, Montez JM, Carroll KM, Darvishzadeh JG et al. (1996) Abnormal splicing of the leptin receptor in diabetic mice. Nature 379: 632-635. doi:10.1038/379632a0. PubMed: 8628397. 8628397

[24] White DW, Tartaglia LA (1996) Leptin and OB-R: body weight regulation by a cytokine receptor. Cytokine Growth Factor Rev 7: 303-309. doi:10.1016/S1359-6101(96)00040-8. PubMed: 9023054. 9023054

[25] Taga T (1996) Gp130, a shared signal transducing receptor component for hematopoietic and neuropoietic cytokines. J Neurochem 67: 1-10. PubMed: 8666978. 8666978 10.1046/j.1471-4159.1996.67010001.x

[26] Haan C, Kreis S, Margue C, Behrmann I (2006) Jaks and cytokine receptors--an intimate relationship. Biochem Pharmacol 72: 1538-1546. doi:10.1016/j.bcp.2006.04.013. PubMed: 16750817. 16750817

[27] White UA, Stephens JM (2011) The gp130 receptor cytokine family: regulators of adipocyte development and function. Curr Pharm Des 17: 340-346. doi:10.2174/138161211795164202. PubMed: 21375496. 21375496 PMC3119891

[28] Bjørbaek C (2009) Central Leptin Receptor Action and Resistance in Obesity. J Invest Med 57: 789-779. PubMed: 20029269. 10.231/JIM.0b013e3181bb0d49 PMC2798145 20029269

[29] Leinninger GM, Myers MG Jr (2008) LRb signals act within a distributed network of leptin-responsive neurones to mediate leptin action. Acta Physiol (Oxf) 192: 49-59. PubMed: 18171429. 18171429 10.1111/j.1748-1716.2007.01784.x

[30] Münzberg H, Huo L, Nillni EA, Hollenberg AN, Bjørbaek C (2003) Role of signal transducer and activator of transcription 3 in regulation of hypothalamic proopiomelanocortin gene expression by leptin. Endocrinology 144: 2121-2131. doi:10.1210/en.2002-221037. PubMed: 12697721. 12697721

[31] Bates SH, Stearns WH, Dundon TA, Schubert M, Tso AW et al. (2004) STAT3 signalling is required for leptin regulation of energy balance but not reproduction. Nature 421: 856-859. 10.1038/nature01388 12594516

[32] Piper ML, Unger EK, Myers MG Jr, Xu AW (2008) Specific physiological roles for signal transducer and activator of transcription 3 in leptin receptor-expressing neurons. Mol Endocrinolohy 22: 751-759. PubMed: 18096691. 10.1210/me.2007-0389 PMC2262173 18096691

[33] Dhillon H, Zigman JM, Ye C, Lee CE, McGovern RA et al. (2006) Leptin directly activates SF1 neurons in the VMH, and this action by leptin is required for normal body-weight homeostasis. Neuron 49: 191-203. doi:10.1016/j.neuron.2005.12.021. PubMed: 16423694. 16423694

[34] Hill JW, Elias CF, Fukuda M, Williams KW, Berglund ED et al. (2010) Direct insulin and leptin action on pro-opiomelanocortin neurons is required for normal glucose homeostasis and fertility. Cell Metab 11: 286-297. doi:10.1016/j.cmet.2010.03.002. PubMed: 20374961. 20374961 PMC2854520

[35] Shanley LJ, Irving AJ, Harvey J (2001) Leptin enhances NMDA receptor function and modulates hippocampal synaptic plasticity. J Neurosci 21: RC186:RC186 PubMed: 11734601. 10.1523/JNEUROSCI.21-24-j0001.2001 PMC6763052 11734601

[36] Irving AJ, Wallace L, Durakoglugil D, Harvey J (2006) Leptin enhances NR2B-mediated N-methyl-D-aspartate responses via a mitogen-activated protein kinase-dependent process in cerebellar granule cells. Neuroscience.138: 1137-1148. doi:10.1016/j.neuroscience.2005.11.042. PubMed: 16413128. 16413128 PMC1613257

[37] Tartaglia LA, Dembski M, Weng X, Deng N, Culpepper J et al. (1995) Identification and expression cloning of a leptin receptor, OB-R. Cell 83: 1263-1271. doi:10.1016/0092-8674(95)90151-5. PubMed: 8548812. 8548812

[38] Huo L, Gamber K, Greeley S, Silva J, Huntoon N, Leng XH, Bjørbaek C (2009) Leptin-dependent Control of Glucose Balance and Locomotor Activity by POMC Neurons. Cell Metab 9: 537-547. doi:10.1016/j.cmet.2009.05.003. PubMed: 19490908. 19490908 PMC2730605

[39] Tong Q, Ye CP, Jones JE, Elmquist JK, Lowell BB (2008) Synaptic release of GABA by AgRP neurons is required for normal regulation of energy balance. Nat Neurosci 11: 998-1000. doi:10.1038/nn.2167. PubMed: 19160495. 19160495 PMC2662585

[40] Novak A, Guo C, Yang W, Nagy A, Lobe CG (2000) Z/EG, a double reporter mouse line that expresses enhanced green fluorescent protein upon Cre-mediated excision. Genesis 28: 147-155. doi:10.1002/1526-968X(200011/12)28:3/4. PubMed: 11105057. 11105057

[41] Qiu J, Bosch MA, Tobias SC, Grandy DK, Scanlan TS et al. (2003) Rapid signaling of estrogen in hypothalamic neurons involves a novel G-protein-coupled estrogen receptor that activates protein kinase C. J Neurosci 23: 9529-9540. PubMed: 14573532. 14573532 10.1523/JNEUROSCI.23-29-09529.2003 PMC6740471

[42] Bjørbaek C, Uotani S, da Silva B, Flier JS (1997) Divergent signaling capacities of long and short isoforms of the leptin receptor. J Biol Chem 272: 32686-32695. doi:10.1074/jbc.272.51.32686. PubMed: 9405487. 9405487

[43] Bjørbaek C, Vik TA, Echwald SM, Yang PY, Vestergaard H et al. (1995) Cloning of a human insulin-stimulated protein kinase (ISPK-1) gene and analysis of coding regions and mRNA levels of the ISPK-1 and the protein phosphatase-1 genes in muscle from NIDDM patients. Diabetes 44: 90-97. doi:10.2337/diab.44.1.90. PubMed: 7813820. 7813820

[44] Huo L, Grill HJ, Bjørbaek C (2006) Divergent regulation of POMC neurons by leptin in the NTS and the arcuate nucleus of the hypothalamus. Diabetes 55: 567-573. doi:10.2337/diabetes.55.03.06.db05-1143. PubMed: 16505217. 16505217

[45] Huo L, Maeng L, Bjørbaek C, Grill HJ (2007) Leptin and the Control of Food Intake: Neurons in the Nucleus of the Solitary Tract (NTS) are Activated by Both Gastric Distension and Leptin. Endocrinology 148: 2189-2197. doi:10.1210/en.2006-1572. PubMed: 17317774. 17317774

[46] Ngwenya LB, Peters A, Rosene DL (2005) Light and electron microscopic immunohistochemical detection of bromodeoxyuridine-labeled cells in the brain: different fixation and processing protocols. J Histochem Cytochem 53: 821-832. doi:10.1369/jhc.4A6605.2005. PubMed: 15995140. 15995140

[47] Fiala JC (2005) Reconstruct: a free editor for serial section microscopy. J Microsc 218: 52-61. doi:10.1111/j.1365-2818.2005.01466.x. PubMed: 15817063. 15817063

[48] Lu J, Fiala JC, Lichtman JW (2009) Semi-automated reconstruction of neural processes from large numbers of fluorescence images. PLOS ONE 4: e5655. doi:10.1371/journal.pone.0005655. PubMed: 19479070. 19479070 PMC2682575

[49] Peters A, Palay SL, Webster HD (1991) The fine structure of the nervous system. New York: Oxford University Press.

[50] Stuart G, Spruston N, Hausser M (2008) In “Dendrites”,. New York: Oxford Press.

[51] Huo L, Münzberg H, Nillni EA, Bjørbaek C (2004) Role of signal transducer and activator of transcription 3 in regulation of hypothalamic trh gene expression by leptin. Endocrinology 145: 2516-2523. doi:10.1210/en.2003-1242. PubMed: 14764629. 14764629

[52] Elmquist JK, Bjørbaek C, Ahima RS, Flier JS, Saper CB (1998) Distributions of leptin receptor isoforms in the rat brain. J Comp Neurol 395: 535-547. doi:10.1002/(SICI)1096-9861(19980615)395:4. PubMed: 9619505. 9619505

[53] Scott MM, Lachey JL, Sternson SM, Lee CE, Elias CF et al. (2009) Leptin targets in the mouse brain. J Comp Neurol 514: 518-532. doi:10.1002/cne.22025. PubMed: 19350671. 19350671 PMC2710238

[54] Kiss JZ, Cassell MD, Palkovits M (1984) Analysis of the ACTH/beta-End/alpha-MSH-immunoreactive afferent input to the hypothalamic paraventricular nucleus of rat. Brain Res 324: 91-99. doi:10.1016/0006-8993(84)90625-5. PubMed: 6097342. 6097342

[55] Mihály E, Fekete C, Tatro JB, Liposits Z, Stopa EG et al. (2000) Hypophysiotropic thyrotropin-releasing hormone-synthesizing neurons in the human hypothalamus are innervated by neuropeptide Y, agouti-related protein, and alpha-melanocyte-stimulating hormone. J Clin Endocrinol Metab 85: 2596-2603. doi:10.1210/jc.85.7.2596. PubMed: 10902813. 10902813

[56] Horvath TL, Naftolin F, Kalra SP, Leranth C (1992) Neuropeptide-Y innervation of beta-endorphin-containing cells in the rat mediobasal hypothalamus: a light and electron microscopic double immunostaining analysis. Endocrinology 131: 2461-2467. doi:10.1210/en.131.5.2461. PubMed: 1425443. 1425443

[57] Jacobowitz DM, O'Donohue TL (1987) alpha-Melanocyte stimulating hormone: immunohistochemical identification and mapping in neurons of rat brain. Proc Natl Acad Sci U S A 75: 6300-6304. PubMed: 366617. 10.1073/pnas.75.12.6300 PMC393169 366617

[58] Bouret SG, Bates SH, Chen S, Myers MG Jr, Simerly RB (2012) Distinct roles for specific leptin receptor signals in the development of hypothalamic feeding circuits. J Neurosci 32: 1244-1252. doi:10.1523/JNEUROSCI.2277-11.2012. PubMed: 22279209. 22279209 PMC3567460

[59] Hentges ST (2007) Synaptic regulation of proopiomelanocortin neurons can occur distal to the arcuate nucleus. J Neurophysiol 97: 3298-3304. doi:10.1152/jn.00051.2007. PubMed: 17360821. 17360821

[60] Liao GY, An JJ, Gharami K, Waterhouse EG, Vanevski F et al. (2012) Dendritically targeted Bdnf mRNA is essential for energy balance and response to leptin. Nat Med 18: 564-571. doi:10.1038/nm.2687. PubMed: 22426422. 22426422 PMC3327556

[61] MacLennan AJ, Neitzel KL, Devlin BK, Garcia J, Hauptman GA et al. (2000) In vivo localization and characterization of functional ciliary neurotrophic factor receptors which utilize JAK-STAT signaling. Neuroscience 99: 761-772. doi:10.1016/S0306-4522(00)90236-7. PubMed: 10974439. 10974439

[62] Stroh T, Sarret P, Tannenbaum GS, Beaudet A (2006) Immunohistochemical distribution and subcellular localization of the somatostatin receptor subtype 1 (sst1) in the rat hypothalamus. Neurochem Res 31: 247-257. doi:10.1007/s11064-005-9013-7. PubMed: 16518576. 16518576

[63] Reyes AR, Levenson R, Berrettini W, Van Bockstaele EJ (2010) Ultrastructural relationship between the mu opioid receptor and its interacting protein, GPR177, in striatal neurons. Brain Res 1358: 71-80. doi:10.1016/j.brainres.2010.08.080. PubMed: 20813097. 20813097 PMC2956578

[64] Treweek JB, Jaferi A, Colago EE, Zhou P, Pickel VM (2009) Electron microscopic localization of corticotropin-releasing factor (CRF) and CRF receptor in rat and mouse central nucleus of the amygdala. J Comp Neurol 512: 323-335. doi:10.1002/cne.21884. PubMed: 19003957. 19003957 PMC2873768

[65] Moult PR, Cross A, Santos SD, Carvalho AL, Lindsay Y et al. (2010) Leptin regulates AMPA receptor trafficking via PTEN inhibition. J Neurosci 30: 4088-4101. doi:10.1523/JNEUROSCI.3614-09.2010. PubMed: 20237279. 20237279 PMC2843829

[66] Grafstein B, Forman DS (1980) Intracellular transport in neurons. Physiol Rev 60: 1167-1283. PubMed: 6159657. 6159657 10.1152/physrev.1980.60.4.1167

[67] Hirokawa N (1998) Kinesin and dynein superfamily proteins and the mechanism of organelle transport. Science 279: 519-526. doi:10.1126/science.279.5350.519. PubMed: 9438838. 9438838

[68] Sickles DW, Stone JD, Friedman MA (1998) Fast axonal transport: a site of acrylamide neurotoxicity? Neurotoxicology 23: 223-251. PubMed: 12224764. 10.1016/s0161-813x(02)00025-6 12224764

[69] O'Malley D, Irving AJ, Harvey J (2005) Leptin-induced dynamic alterations in the actin cytoskeleton mediate the activation and synaptic clustering of BK channels. FASEB J 19: 1917-1919. PubMed: 16166199. 16166199 10.1096/fj.05-4166fje

[70] O'Malley D, MacDonald N, Mizielinska S, Connolly CN, Irving AJ et al. (2007) Leptin promotes rapid dynamic changes in hippocampal dendritic morphology. Mol Cell Neurosci 35: 559-572. doi:10.1016/j.mcn.2007.05.001. PubMed: 17618127. 17618127 PMC1995039

[71] McKinney RA (2010) Excitatory amino acid involvement in dendritic spine formation, maintenance and remodelling. J Physiol 588: 107-116. doi:10.1113/jphysiol.2009.178905. PubMed: 19933758. 19933758 PMC2821552

[72] Goldstein LS, Yang Z (2000) Microtubule-based transport systems in neurons: the roles of kinesins and dyneins. Annu Rev Neurosci 23: 39-71. doi:10.1146/annurev.neuro.23.1.39. PubMed: 10845058. 10845058

[73] Ng DC, Lin BH, Lim CP, Huang G, Zhang T et al. (2006) Stat3 regulates microtubules by antagonizing the depolymerization activity of stathmin. J Cell Biol 172: 245-257. doi:10.1083/jcb.200503021. PubMed: 16401721. 16401721 PMC2063554

[74] Verma NK, Dourlat J, Davies AM, Long A, Liu WQ et al. (2009) STAT3-stathmin interactions control microtubule dynamics in migrating T-cells. J Biol Chem 284: 12349-12362. doi:10.1074/jbc.M807761200. PubMed: 19251695. 19251695 PMC2673303

[75] Tahirovic S, Bradke F (2009) Neuronal polarity. Cold Spring Harb Perspect Biol 1: a001644. doi:10.1101/cshperspect.a001644. PubMed: 20066106. 20066106 PMC2773638

[76] Nicolas CS, Peineau S, Amici M, Csaba Z, Fafouri A et al. (2012) The Jak/STAT pathway is involved in synaptic plasticity. Neuron 73: 374-390. doi:10.1016/j.neuron.2011.11.024. PubMed: 22284190. 22284190 PMC3268861

[77] Flynn KC (2013) The cytoskeleton and neurite initiation. Bioarchitecture 3(4): ([MedlinePgn:]) PubMed: 24002528. 10.4161/bioa.26259 PMC4201609 24002528

[78] Stowell JN, Craig AM (1999) Axon/dendrite targeting of metabotropic glutamate receptors by their cytoplasmic carboxy-terminal domains. Neuron 22: 525-536. doi:10.1016/S0896-6273(00)80707-2. PubMed: 10197532. 10197532

[79] Francesconi A, Duvoisin RM (2002) Alternative splicing unmasks dendritic and axonal targeting signals in metabotropic glutamate receptor 1. J Neurosci 22: 2196-2205. PubMed: 11896159. 11896159 10.1523/JNEUROSCI.22-06-02196.2002 PMC6758268

[80] Setou M, Seog DH, Tanaka Y, Kanai Y, Takei Y et al. (2002) Glutamate-receptor-interacting protein GRIP1 directly steers kinesin to dendrites. Nature 417: 83-87. doi:10.1038/nature743. PubMed: 11986669. 11986669

[81] Rivera JF, Ahmad S, Quick MW, Liman ER, Arnold DB (2003) An evolutionarily conserved dileucine motif in Shal K+ channels mediates dendritic targeting. Nat Neurosci 6: 243-250. doi:10.1038/nn1020. PubMed: 12592409. 12592409

[82] Xu X, He C, Zhang Z, Chen Y (2006) MKLP1 requires specific domains for its dendritic targeting. J Cell Sci 119: 452-458. doi:10.1242/jcs.02750. PubMed: 16418225. 16418225

[83] Gray EG (1959) Electron microscopy of synaptic contacts on dendrite spines of the cerebral cortex. Nature 183: 1592-1593. doi:10.1038/1831592a0. PubMed: 13666826. 13666826

[84] Klemann CJ, Roubos EW (2011) The gray area between synapse structure and function-Gray's synapse types I and II revisited. Synapse 65: 1222-1230. doi:10.1002/syn.20962. PubMed: 21656572. 21656572

[85] Pinto S, Roseberry AG, Liu H, Diano S, Shanabrough M et al. (2004) Rapid rewiring of arcuate nucleus feeding circuits by leptin. Science 304: 110-115. doi:10.1126/science.1089459. PubMed: 15064421. 15064421

