# Correction: Somato-Dendritic Localization and Signaling by Leptin Receptors in Hypothalamic POMC and AgRP Neurons

**DOI:** 10.1371/annotation/a3ef8765-266f-4e4b-8370-264d6f6f301e

**Published:** 2013-12-05

**Authors:** Sangdeuk Ha, Scott Baver, Lihong Huo, Adriana Gata, Joyce Hairston, Nicholas Huntoon, Wenjing Li, Thompson Zhang, Elizabeth J. Benecchi, Maria Ericsson, Shane T. Hentges, Christian Bjørbæk

This paper was republished on December 5. The legend for Figure 9 was erroneously omitted from the figure and included in the text of the article. Please see the corrected Figure 9 with figure legend. 

