# Supplementary figures and images for: Somato-Dendritic Localization and Signaling by Leptin Receptors in Hypothalamic POMC and AgRP Neurons
Source: PLoS One. 2013 Oct 29;8(10):e77622. doi: 10.1371/journal.pone.0077622 (PMC3812230; doi:10.1371/journal.pone.0077622)

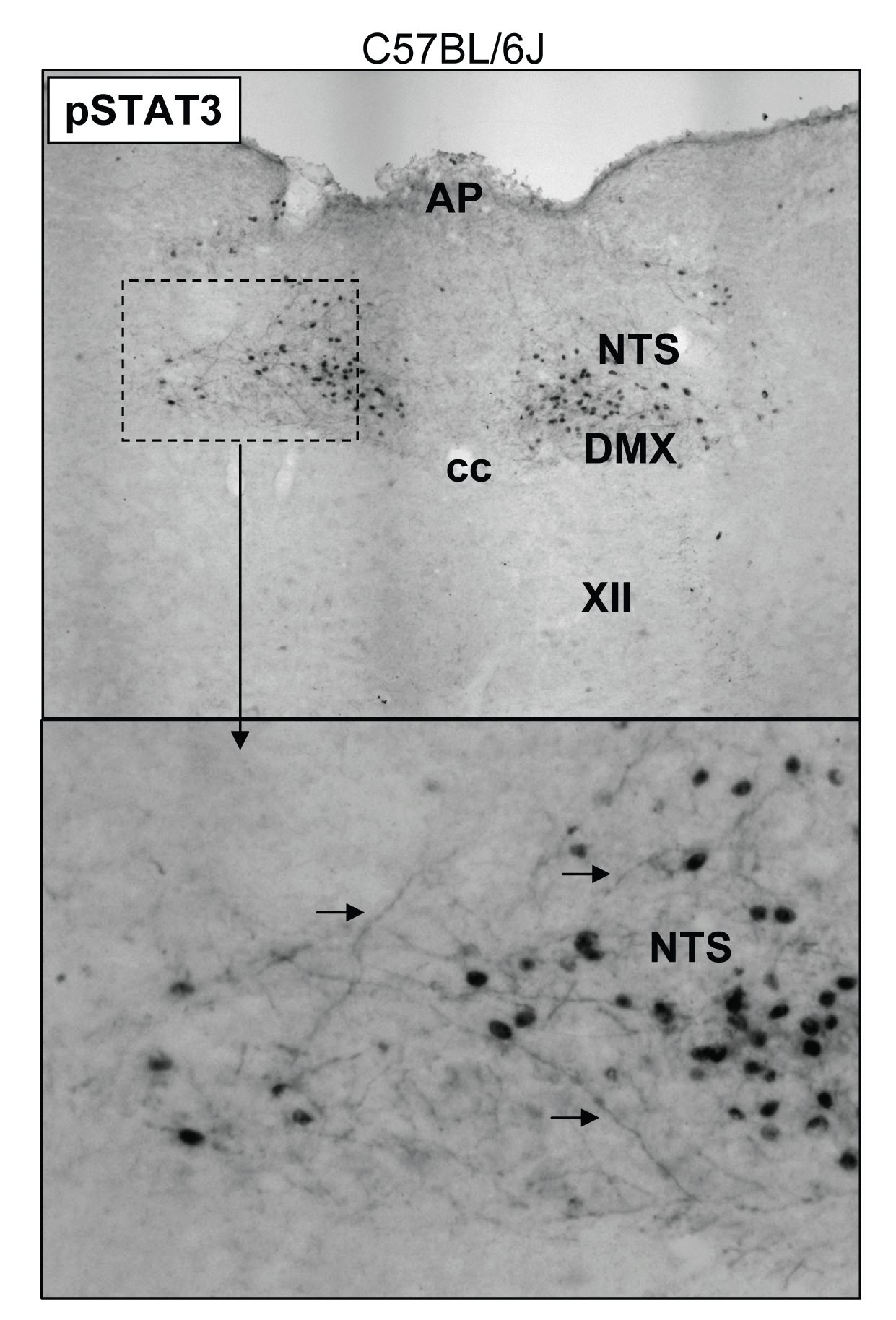

Supplement: Figure S1 — Leptin activates STAT3 phosphorylation in neuronal fiber processes within the NTS of C57BL/6J mice. Shown are light microscopy (LM) images of phospho-STAT3IR (DAB) in coronal brain sections of the hindbrain from 8 weeks old wild type C57BL/6J male mice. Animals were given leptin (5 mg/kg, ip) and sacrificed after 30 minutes. Top: pSTAT3 IR is found within the NTS at the level of the area postrema (AP). The bottom image shows high magnification of stippled box from top. Arrows identify some of many pSTAT3 IR neuronal processes. XII: Hypoglossal nerve; NTS: nucleus of the solitary tract; DMX: dorsal motor nucleus of the vagus nerve; cc: central canal, AP: area postrema. (TIF) [file pone.0077622.s001.tif]

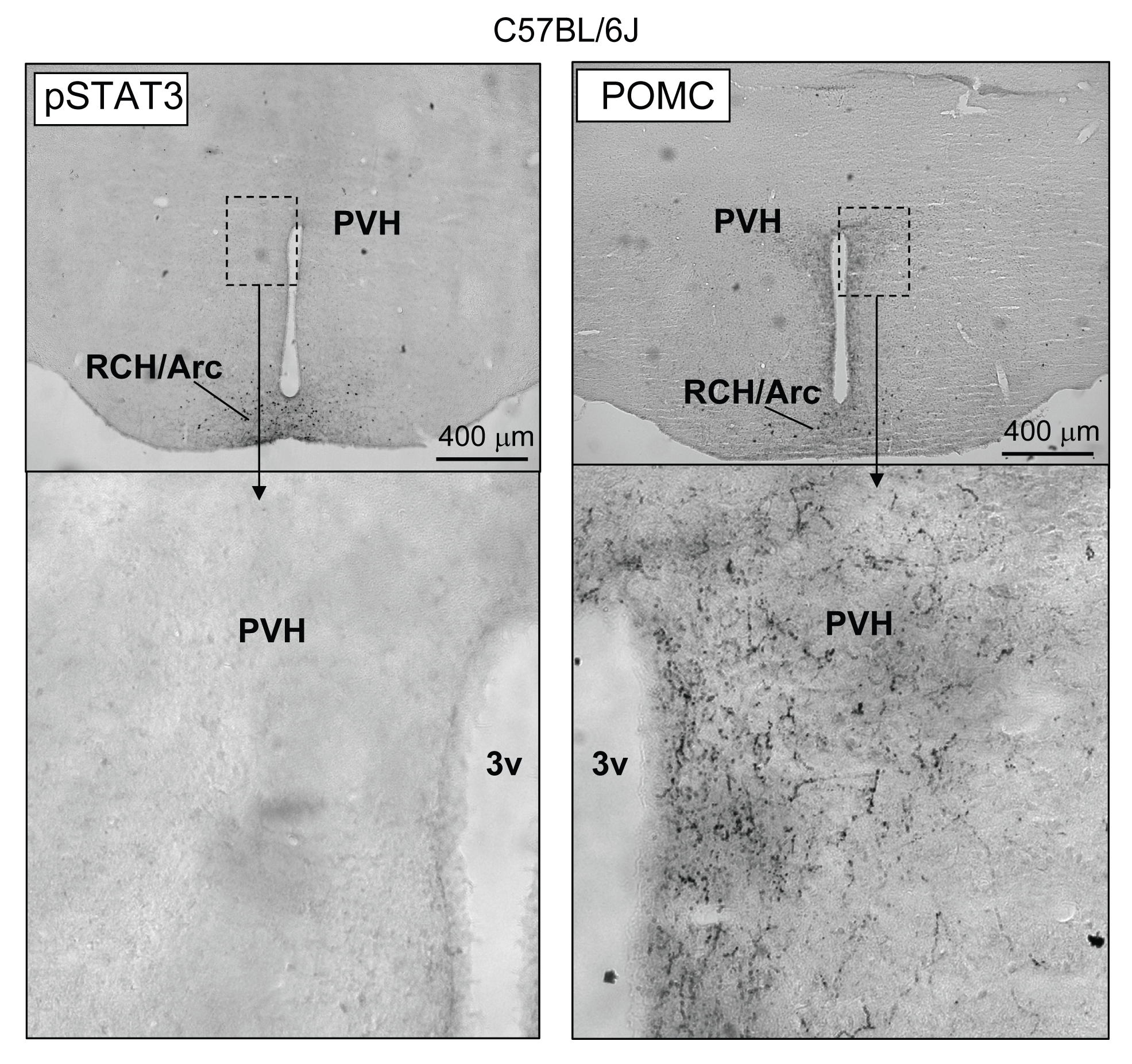

Supplement: Figure S2 — Leptin does not induce STAT3 phosphorylation within the PVH, a major axonal target zone of leptin-responsive POMC neurons. Left: pSTAT3 IR is present in the RCH/Arc region of the anterior hypothalamus, but importantly, not in the PVH of leptin-treated (5 mg/kg i.p., 30 minutes) C57BL/6J mice. Bottom: High-magnification microphotograph demonstrating lack of pSTAT3 IR fibers (and nuclei) in the PVH. Right: Many POMC IR nuclei are found in the RCH/Arc. Bottom: Dense networks of neuronal (axonal) POMC fibers are observed in PVH. RCH: retrochiasmatic area; Arc: arcuate; 3v: 3rd ventricle; PVH: paraventricular hypothalamic nucleus. (TIF) [file pone.0077622.s002.tif]

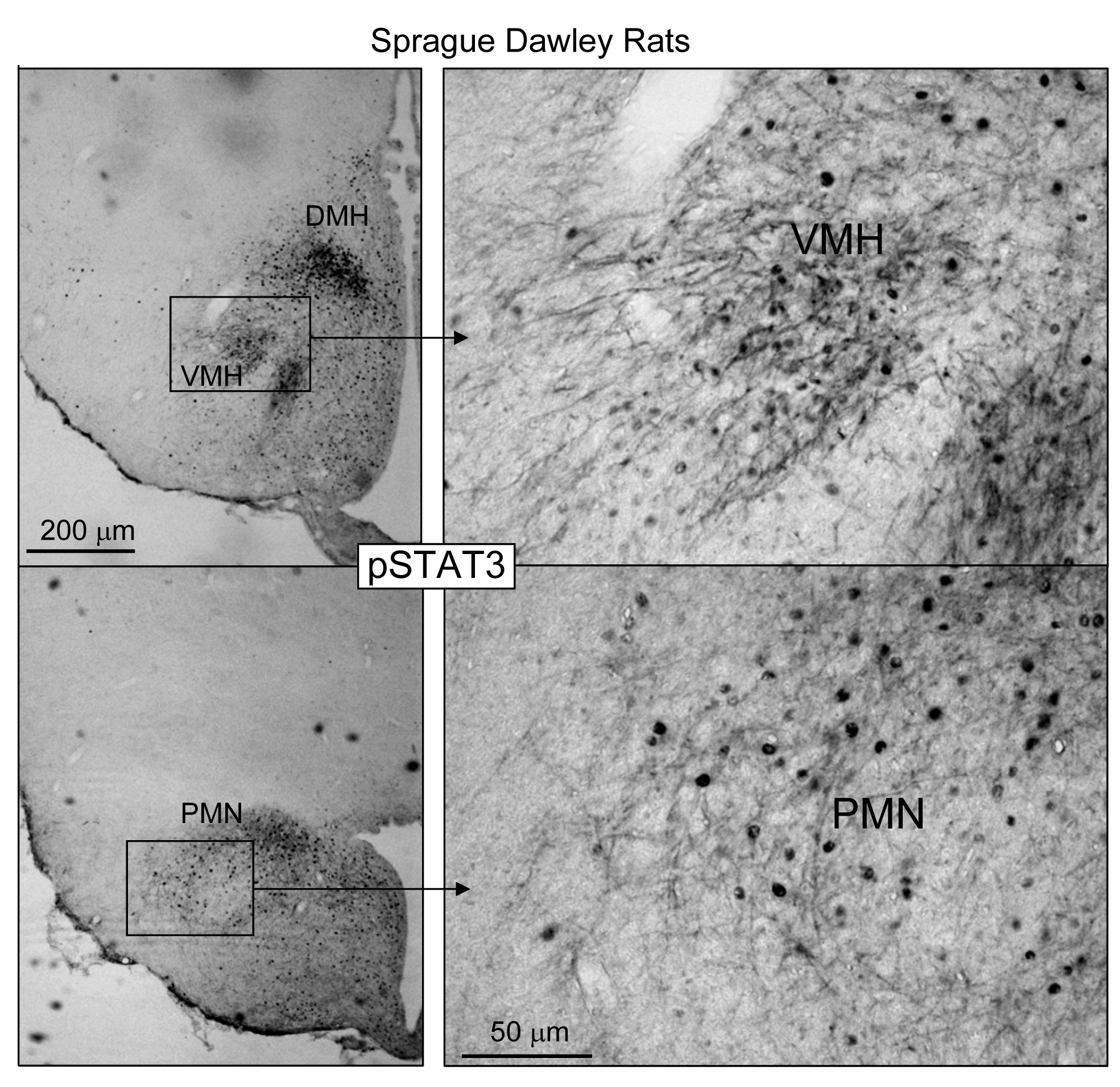

Supplement: Figure S3 — Leptin activates STAT3 phosphorylation in neuronal fiber processes in hypothalamic nuclei of Sprague Dawley rats. Left: Shown are light microscopy (LM) images of phospho-STAT3 IR (DAB) in coronal brain sections of the mediobasal hypothalamus from Sprague Dawley rats. Rats were given leptin (5 mg/kg, ip) for 45 minutes. . Right: High-magnification microphotographs of boxes in left column. Robust pSTAT3 IR is found in fibers within the VMH and PMN. (TIF) [file pone.0077622.s003.tif]

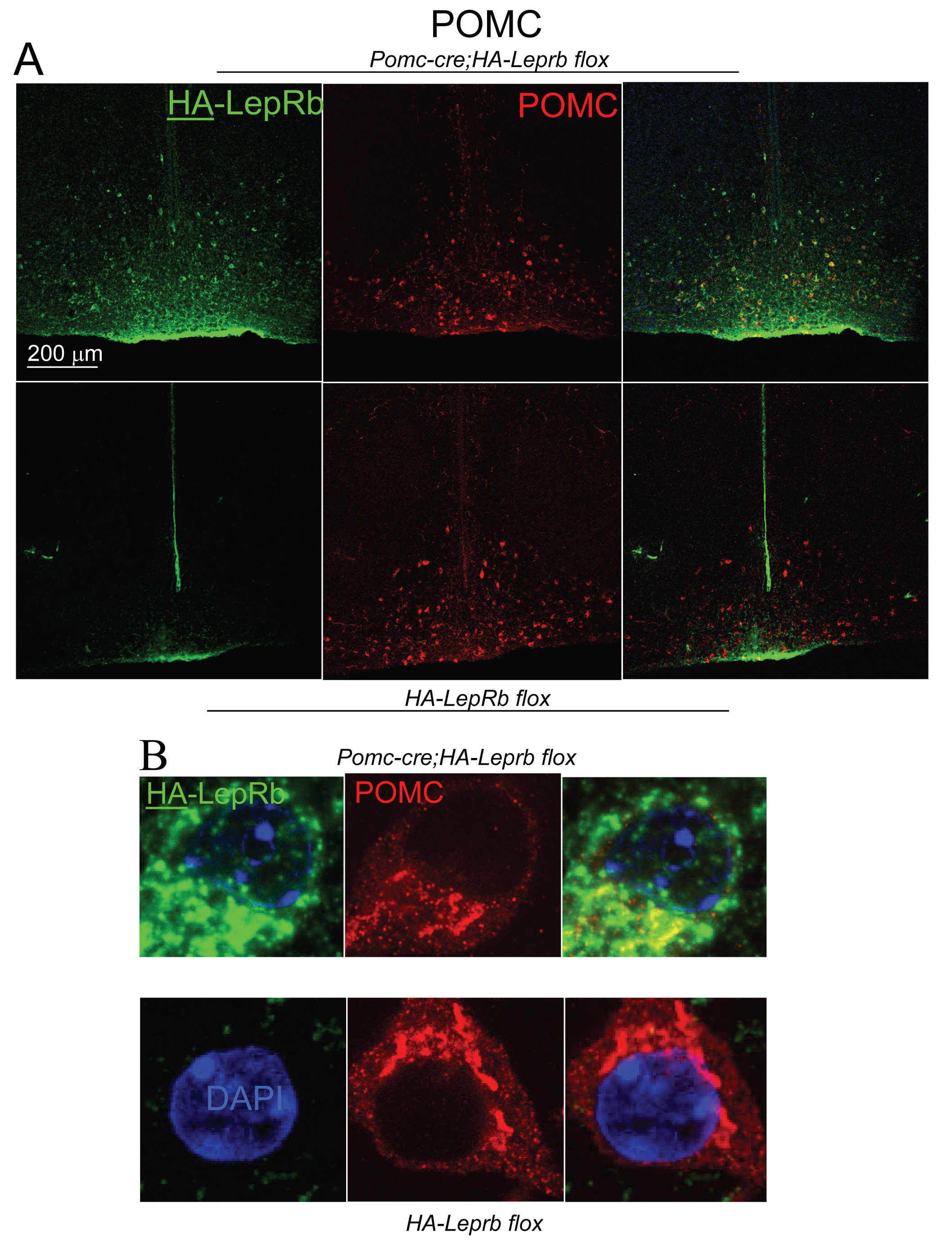

Supplement: Figure S4 — Expression of HA-tagged LepRb in hypothalamic POMC neurons. A. CLSM of POMC neurons (red (POMC-polypeptide IR)) and HA-LepRb (green (HA IR)) in a hypothalamic brain section from a Pomc-cre;HA-Leprb flox mouse (top row) and a negative control section from a HA-Leprb flox mouse (bottom row). Some non-specific HA IR (green) is observed along the lining of the 3rd ventricle and at the base of the Arc in the control section. Shown are single confocal planes. B. Top row: Example of a POMC soma (red) co-expressing HA-LepRb (green) in a Pomc-cre;HA-Leprb flox mouse. DAPI fluorescence (blue) identifies the nucleus. Bottom row: Example of a POMC neuron that does not express HA-LepRb in a HA-Leprb flox control mouse. Shown are single confocal planes. (TIF) [file pone.0077622.s004.tif]

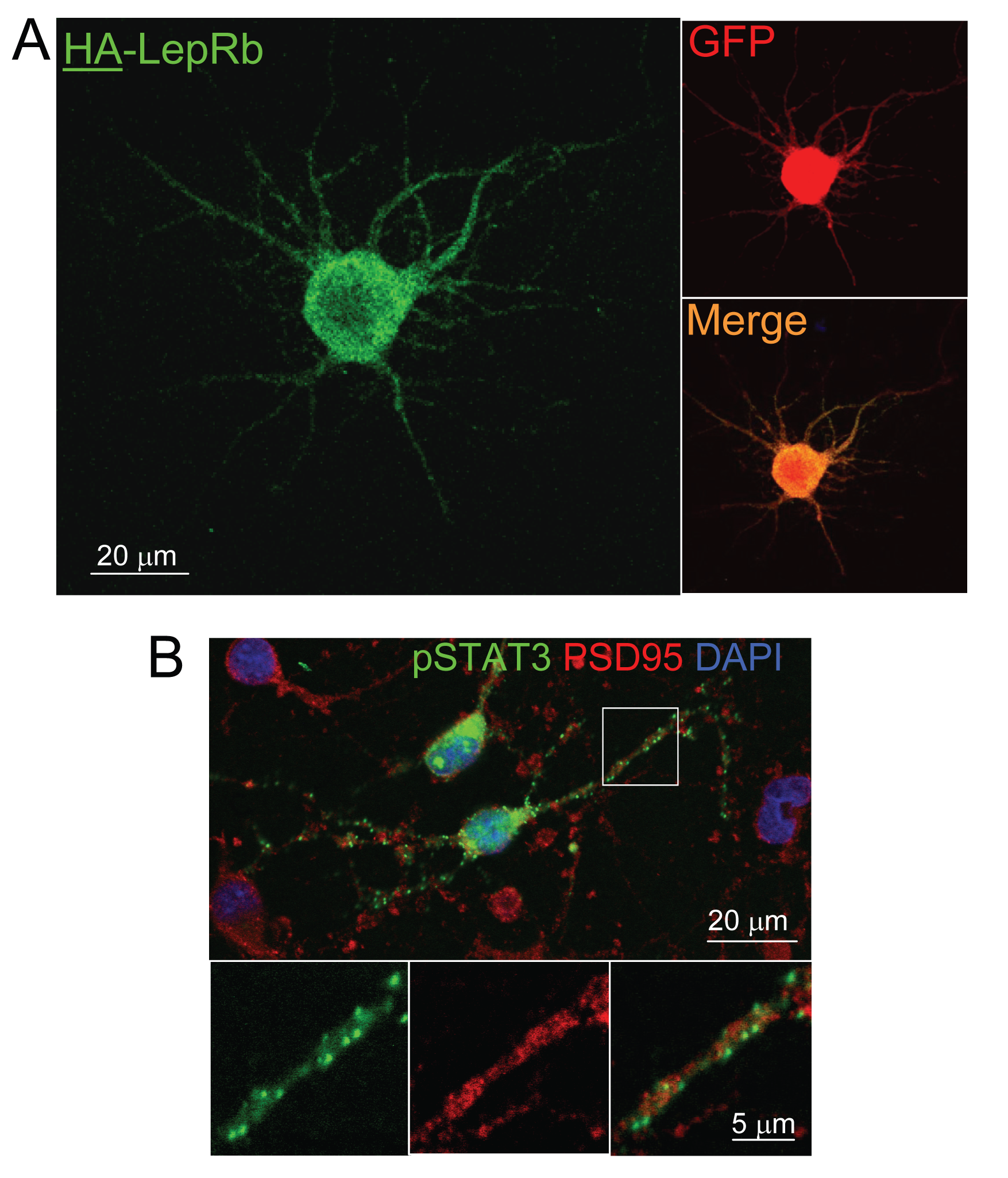

Supplement: Figure S5 — Localization of leptin receptors and activation of STAT3 in neuronal fibers of transfected primary neurons. A. Primary hypothalamic neurons were co-transfected with plasmids encoding HA-tagged LepRb and GFP, and subjected to immunocytochemistry (ICC) for HA (green) and GFP (red). Leptin receptors are expressed in the soma and in fibers. Shown are collapsed confocal Z-stack sections B. As in A., neurons were transfected with plasmids encoding HA-LepRb. At DIV 12, cells were treated 100 nM leptin for 20 min and fixed. Slides were then subjected to ICC for pSTAT3 (green) and PSD95, a dendritic protein marker (red). DAPI (blue) was included to label nuclei. Top: Two neurons exhibit pSTAT3 IR in the soma and fibers. Bottom: Enlargement of box in top image showing punctate pSTAT3 staining in PSD95 positive fibers. Shown are single confocal planes. DIV: days in vitro. (TIF) [file pone.0077622.s005.tif]

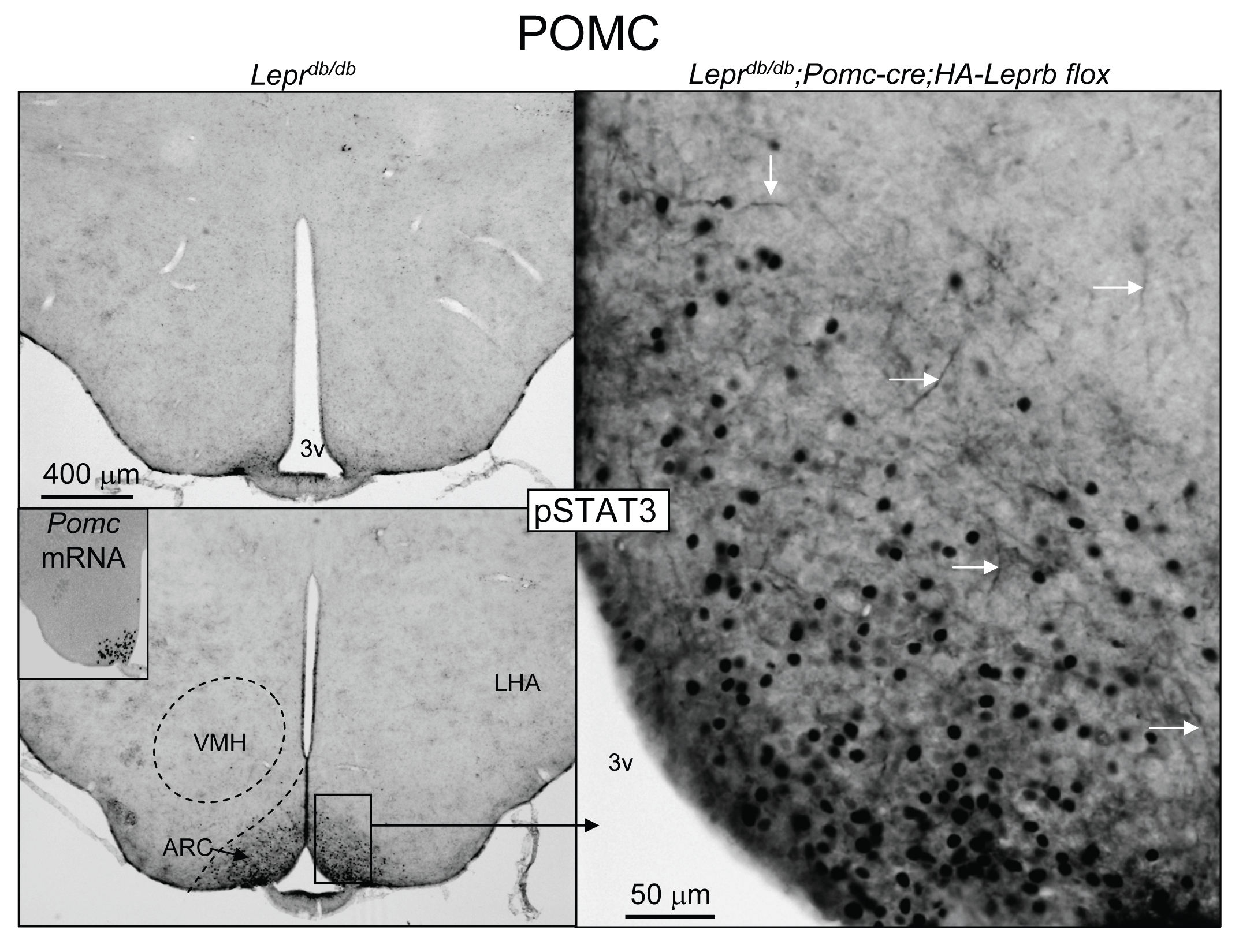

Supplement: Figure S6 — Leprdb/db ;Pomc-cre;HA-LepRbflox mice express pSTAT3 in neuronal processes of POMC neurons. Top Left: LM shows lack of pSTAT3 IR (DAB) in the mediobasal hypothalamus of a leptin-treated (5 mg/kg, ip, 20 min) obese Lepr db/db control mouse. Bottom Left: pSTAT3 IR in the arcuate (ARC), but not the VMH or LHA of obese Leprdb/db;Pomc-cre;HA-LepRb flox mice consistent with the targeting of HA-LepRb to POMC neurons. Insert: For comparison, the anatomical localization of POMC neurons is shown by in situ hybridization for Pomc mRNA. Right: Enlargement of box. Many positive pSTAT3 fibers are shown (arrows). 3v: 3rd ventricle; Arcuate: hypothalamic arcuate nucleus; VMH: ventromedial hypothalamic nucleus; LHA: lateral hypothalamic area. (TIF) [file pone.0077622.s006.tif]

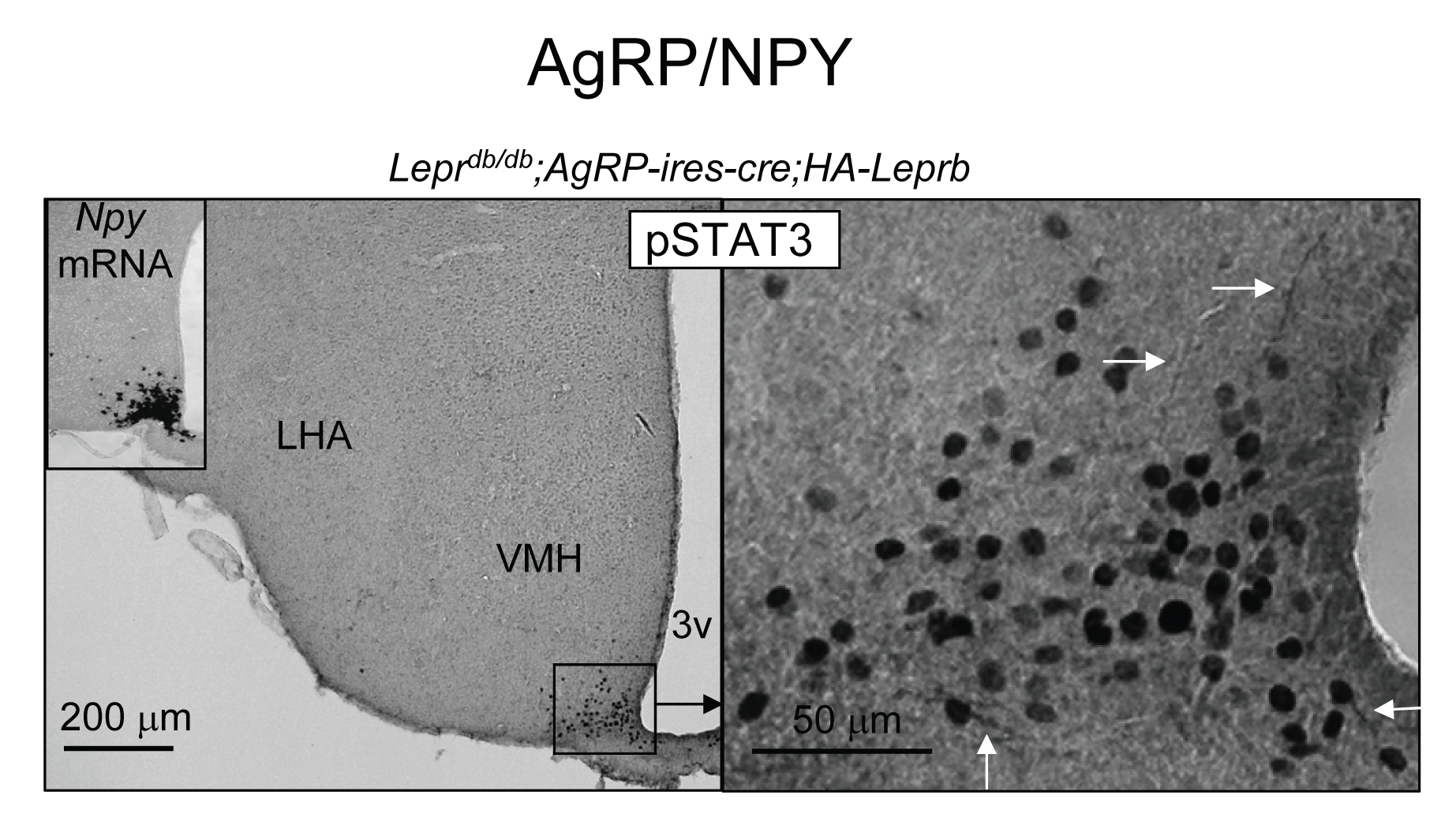

Supplement: Figure S7 — Leprdb/db ;Pomc-cre;HA-LepRbflox mice express pSTAT3 in neuronal processes of AgRP neurons. Left: LM shows pSTAT3 IR (DAB) in the mediobasal hypothalamus of a leptin-treated (5 mg/kg, ip, 20 min) obese Leprdb/db;Pomc-cre;HA-LepRb flox mouse. pSTAT3 IR is found in the medial arcuate (ARC), but not in the VMH or LHA, consistent with targeting of HA-LepRb expression to AgRP neurons. Insert: For comparison, the anatomical localization of AgRP/NPY neurons is shown by in situ hybridization for Npy mRNA. Right: Enlargement of box. Several pSTAT3 IR fibers are visible (arrows). 3v: 3rd ventricle; VMH: ventromedial hypothalamic nucleus; LHA: lateral hypothalamic area. (TIF) [file pone.0077622.s007.tif]

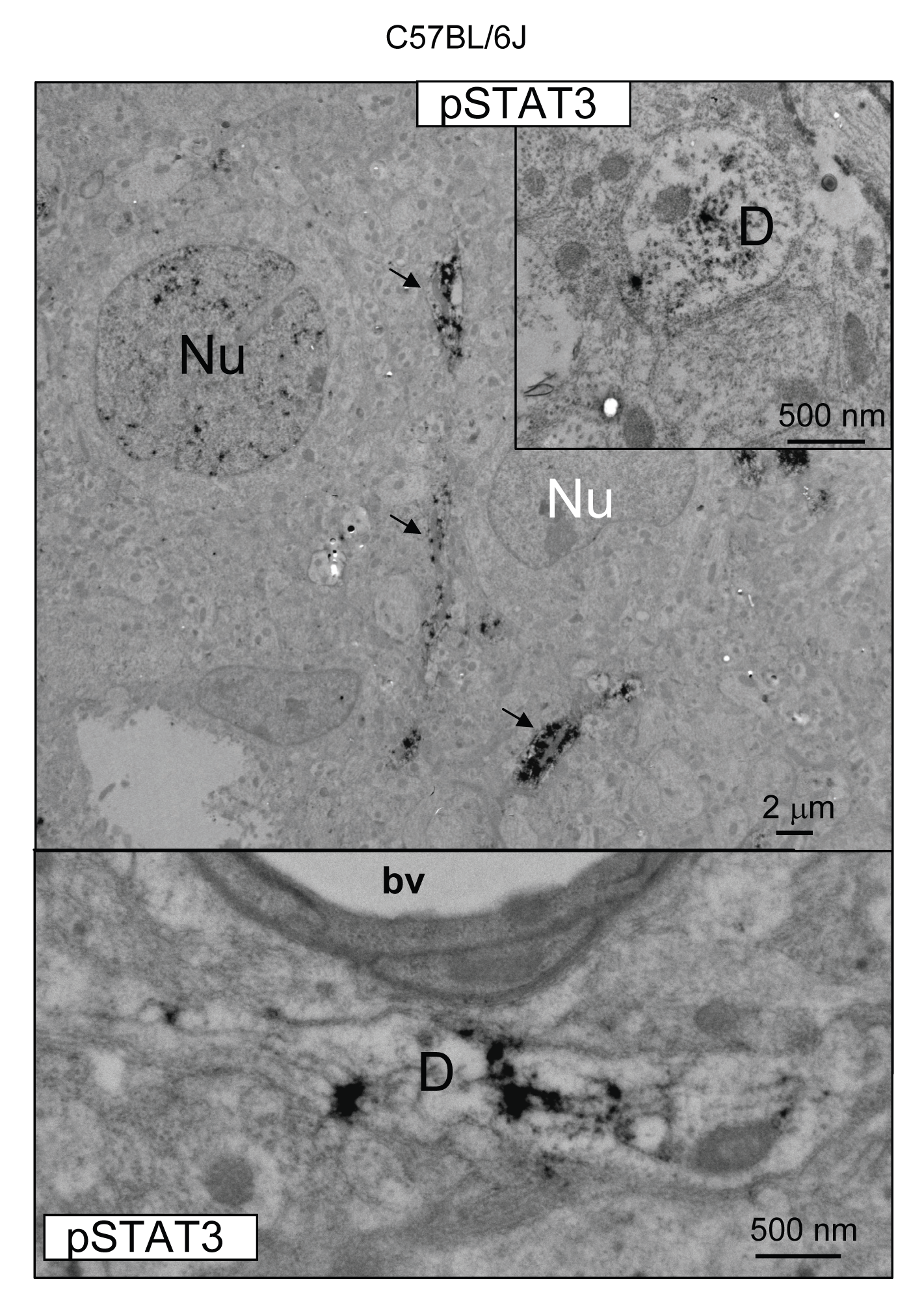

Supplement: Figure S8 — Activation of STAT3 phosphorylation by leptin in dendrites of arcuate hypothalamic neurons of wild type C57BL/6J mice. Immuno-EM for pSTAT3 (DAB) in the arcuate hypothalamic nucleus of a leptin-treated (4 mg/kg, ip, 15 min) wild type C57BL/6J mouse. Top: A pSTAT3 IR neuronal nucleus is labeled with “Nu” (black text) and several pSTAT3 IR dendrites are indicated with black arrows. A pSTAT3 IR negative neuronal nucleus is labeled “Nu” in white text. Insert: A cross sections of a pSTAT3 IR dendritic shaft. Bottom: pSTAT3 IR dendrite in the photographic plane. Nu; nucleus; D: dendrite; bv: blood microvessel. (TIF) [file pone.0077622.s008.tif]

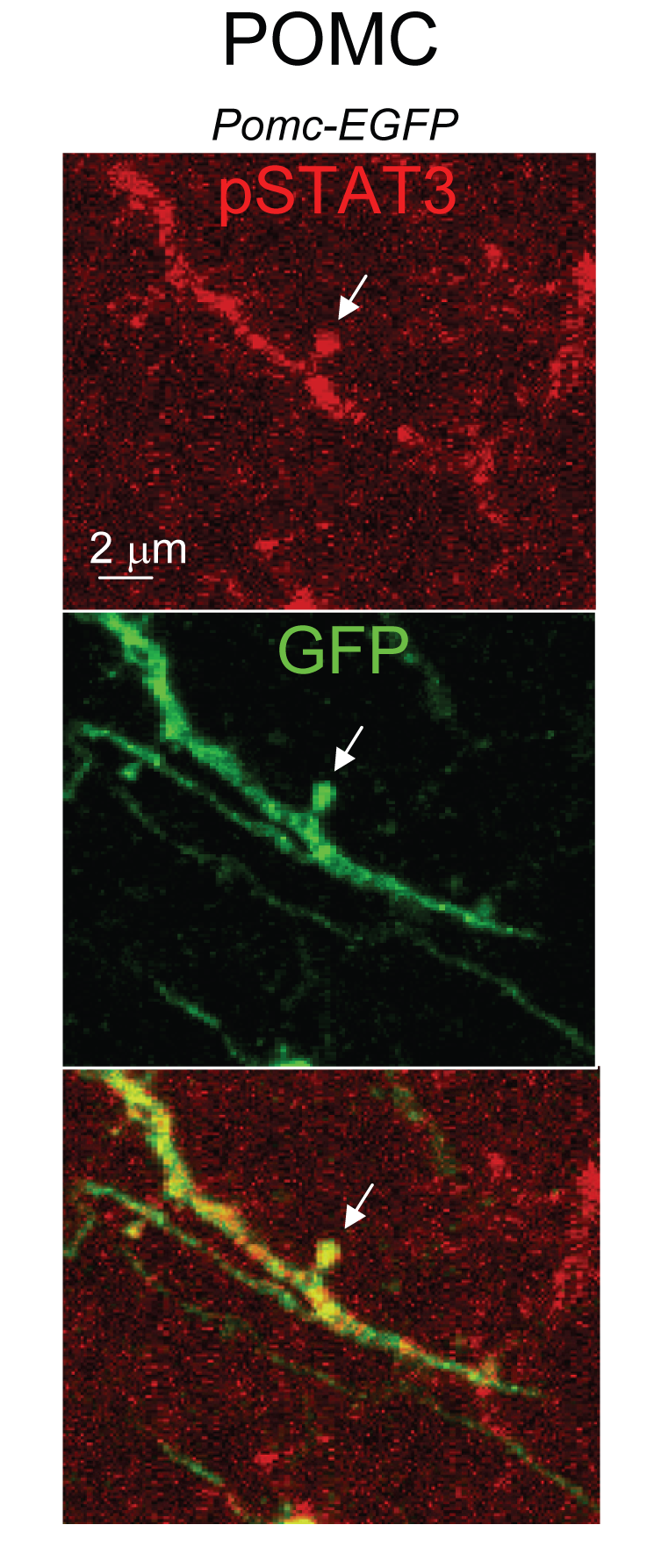

Supplement: Figure S9 — Activation of STAT3 phosphorylation by leptin in dendritic spines of POMC neurons. A Pomc-EGFP mouse was given leptin (5 mg/kg, ip, 30 min). IHC and CLSM was applied to visualize pSTAT3 IR (red) and EGFP-epifluorescence (green) in a brain section of the arcuate nucleus of the hypothalamus. All images are single confocal planes. The arrow depicts a dendritic spine-like structure. (TIF) [file pone.0077622.s009.tif]
